# A novel strategy for improving watermelon resistance to cucumber green mottle mosaic virus by exogenous boron application

**DOI:** 10.1111/mpp.13234

**Published:** 2022-06-07

**Authors:** Xinyue Bi, Huiyan Guo, Xiaodong Li, Lijiao Zheng, Mengnan An, Zihao Xia, Yuanhua Wu

**Affiliations:** ^1^ Liaoning Key Laboratory of Plant Pathology, College of Plant Protection Shenyang Agricultural University Shenyang China; ^2^ Centre for Biological Disaster Prevention and Control National Forestry and Grassland Administration Shenyang China; ^3^ Xinmin City Agricultural Technology Extension Centre Shenyang China

**Keywords:** CGMMV, differentially expressed genes, exogenous boron, transcriptome analysis, virus‐induced gene silencing, watermelon blood flesh disease, watermelon fruit

## Abstract

The molecular mode controlling cucumber green mottle mosaic virus (CGMMV)‐induced watermelon blood flesh disease (WBFD) is largely unknown. In this study, we have found that application of exogenous boron suppressed CGMMV infection in watermelon fruit and alleviated WBFD symptoms. Our transcriptome analysis showed that the most up‐regulated differentially expressed genes (DEGs) were associated with polyamine and auxin biosynthesis, abscisic acid catabolism, defence‐related pathways, cell wall modification, and energy and secondary metabolism, while the down‐regulated DEGs were mostly involved in ethylene biosynthesis, cell wall catabolism, and plasma membrane functions. Our virus‐induced gene silencing results showed that silencing of *SPDS* expression in watermelon resulted in a higher putrescine content and an inhibited CGMMV infection correlating with no WBFD symptoms. *SBT* and *TUBB1* were also required for CGMMV infection. In contrast, silencing of *XTH23* and *PE/PEI7* (low‐level lignin, cellulose and pectin) and *ATPS1* (low‐level glutathione) promoted CGMMV accumulation. Furthermore, *RAP2‐3*, *MYB6*, *WRKY12*, *H2A*, and *DnaJ11* are likely to participate in host antiviral resistance. In addition, a higher (spermidine + spermine):putrescine ratio, malondialdehyde content, and lactic acid content were responsible for fruit decay and acidification. Our results provide new knowledge on the roles of boron in watermelon resistance to CGMMV‐induced WBFD. This new knowledge can be used to design better control methods for CGMMV in the field and to breed CGMMV resistant watermelon and other cucurbit crops.

## Graphical abstract

Boron inhibits CGMMV infection in watermelon fruits, probably though regulating the expression of *SPDS*, *SBT*, *TUBB1*, *RAP2‐3*, *MYB6*, *WRKY12*, *H2A*, *XTH23*, *PE/PEI7*, *DnaJ11*, and *ATPS1* in various physiological and biochemical pathways.

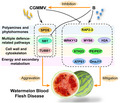

## 1 INTRODUCTION

Watermelon (*Citrullus lanatus*) is a popular and economically important crop worldwide (Guo et al., 2019). China is currently the largest watermelon‐growing country, accounting for about 61% of the global watermelon production in 2019 (http://faostat.fao.org/). Watermelon production in many countries is threatened by various abiotic and biotic stresses, including many plant‐infecting viruses. Cucumber green mottle mosaic virus (CGMMV) is an RNA virus in the genus *Tobamovirus*. CGMMV can infect many cucurbit crops, including watermelon, and cause severe watermelon blood fresh disease (WBFD) in watermelon fruits (Dombrovsky et al., 2017; Li et al., 2017, 2020). The CGMMV‐infected watermelon fruits often show spongy rot, discolouration, and acidification, resulting in significant economic losses (Dombrovsky et al., 2017; Li et al., 2020).

CGMMV was first reported in cucumber in the UK followed by more than 20 countries in Europe, Asia, and South America (Dombrovsky et al., 2017). In 2007, the Agricultural Ministry of China declared CGMMV as a plant quarantine pest that could potentially threat the production of cucurbit crops in China (http://www.moa.gov.cn/). In recent years, several research laboratories have analysed the effects of CGMMV infection on gene expression in watermelon plants (Li et al., 2017, 2020; Sun et al., 2019). However, how CGMMV infection affects watermelon fruit growth and development remained largely unknown, due mainly to the long watermelon growth period and lack of an efficient assay method.

Boron is an essential micronutrient for plant growth and development (Brdar‐Jokanovi, 2020; Camacho‐Cristóbal et al., 2011). The amount of boron in soil is relatively high and the difference between boron deficiency and toxicity is very small. Currently, several factors, including soil pH, texture, and climatic condition, are known to affect boron uptake by plants (Brdar‐Jokanovi, 2020; Shorrocks, 1997). Many reports have shown that boron deficiency can alter various plant physiological processes (Brdar‐Jokanovi, 2020; Camacho‐Cristóbal, Rexach, et al., 2008; Camacho‐Cristóbal et al., 2011; Eggert & Von Wirén, 2017), alter polyamine biosynthesis (Camacho‐Cristóbal et al., 2005), and decrease the expression of several cell wall modification‐associated enzymes while increasing the production of actin and tubulin in *Arabidopsis* roots (Camacho‐Cristóbal, Herrera‐Rodríguez, et al., 2008; Yu et al., 2001). To minimize the damage caused by inadequate boron, plants respond with the synthesis of various hormones (Eggert & Von Wirén, 2017) and activation of signal transduction, stress responses (Lu et al., 2015), and the glycolytic pathway (Zhou et al., 2014). Boron deficiency can be prevented through exogenous boron application to improve crop yield and quality as well as to inhibit plant diseases (Gupta et al., 2017). To date, several research laboratories have demonstrated the relationships between boron homeostasis and virus infection in plants. For example, application of exogenous boron can alleviate disease symptoms caused by tobacco mosaic virus (TMV) or belladonna mottle virus in tobacco plants (Ananthakrishnan et al., 1989; Shimomura, 1982). Moreover, the grapevine Pinot Gris virus (GPGV)‐induced disease symptoms are similar to that induced by boron deficiency, and the expressions of boron homeostasis‐associated genes were affected by both GPGV infection and boron deficiency (Buoso et al., 2020).

Because watermelon is recalcitrant to genetic transformation, analyses of gene functions in this plant are hindered. To circumvent this obstacle, we decided to investigate the molecular mechanisms controlling CGMMV infection in watermelon fruits through reverse genetics using a CGMMV‐based virus‐induced gene silencing (VIGS) pV190 vector as described previously (Lange et al., 2013; Liu et al., 2020). Currently, two VIGS vectors (i.e., the CGMMV‐based pV190 vector and a tobacco rattle virus [TRV]‐based vector) have been reported to be effective in gene silencing in cucurbits (Fang et al., 2021; Liu et al., 2020). In this study, we found that the pV190 vector‐induced gene silencing in watermelon fruits lasted more than 54 days and thus is suitable for studies of CGMMV infection in watermelon fruits and possibly other cucurbit crops.

**FIGURE 1:**
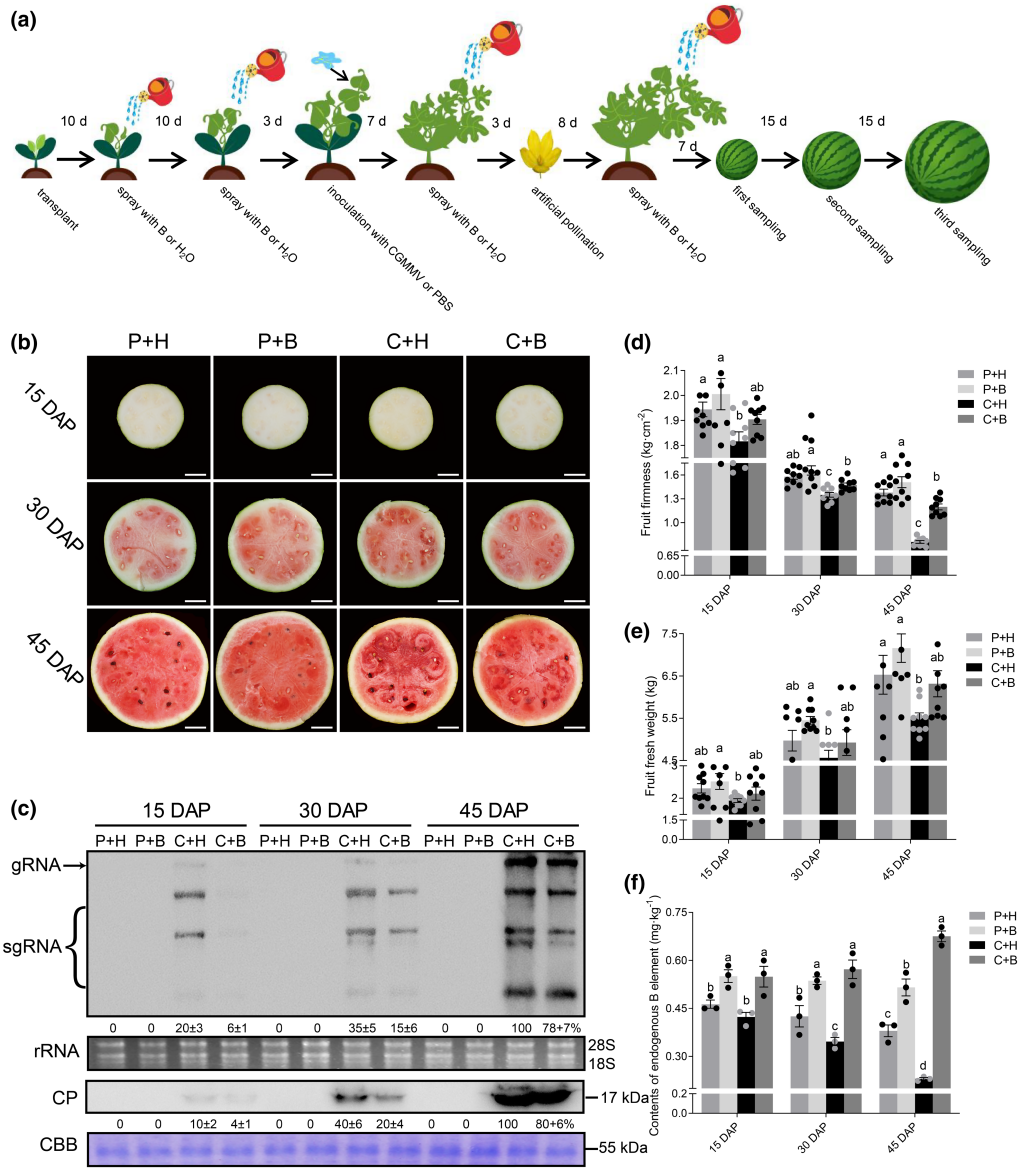
CGMMV‐induced symptoms in watermelon fruits and virus accumulation. (a) Flowchart of CGMMV inoculation, water or boron application, and sampling. (b) Watermelon blood flesh disease symptoms in watermelon fruits from the phosphate‐buffered saline (PBS, P) + water (H)‐, P + boron (B)‐, CGMMV (C) + H‐ or C + B‐treated plants at 15, 30, and 45 days after pollination (DAP), respectively. (c) Northern blot and western blot analyses of CGMMV RNA and coat protein (CP) accumulation in watermelon fruits, respectively. The rRNA gel and the Coomassie brilliant blue (CBB)‐stained gel are used to show sample loadings. (d–f) Measurements of fruit firmness, mean weight of nine fruits per treatment, and mean endogenous boron content (*n* = 3) are shown. The results are presented as the mean ± *SD*. Statistical significance between two different treatments was determined using one‐way analysis of variance followed by Duncan's multiple comparison test. Different lowercase letters indicate statistically significant differences between treatments (*p* < 0.05). Scale bar = 5 cm.

CGMMV‐induced WBFD can significantly affect watermelon yield and quality. Because many reports have indicated that CGMMV infection and boron deficiency cause similar alterations in plant hormone signalling, disease resistance, energy metabolism, and cell wall and membrane functions (Brdar‐Jokanovi, 2020; Li et al., 2017, 2020), we speculated that boron might have a role in watermelon resistance to CGMMV infection. To test this hypothesis, we inoculated watermelon plants with CGMMV and sprayed them with a boron solution or water as described in Figure 1a followed by RNA sequencing (RNA‐Seq) and weighted correlation network analysis (WGCNA). The results showed that the differentially expressed genes (DEGs) found between treatments are mainly involved in polyamine synthesis, hormone regulation, signal transduction, disease resistance, cell wall and plasma membrane functions, and energy and secondary metabolism. To further explore the functions of these DEGs in CGMMV infection, we silenced the expression of 20 selected DEGs in watermelon plants through VIGS using the CGMMV‐based vector. We found that *SPDS*, *SBT*, and *TUBB1* were involved in CGMMV infection and WBFD symptom induction, while *RAP2‐3*, *MYB6*, *WRKY12*, *H2A*, *XTH23*, *PE/PEI7*, *DnaJ11*, and *ATPS1* were likely to participate in watermelon resistance to CGMMV infection. The contents of many metabolites, including polyamines, celluloses, pectins, malondialdehyde (MDA), and lactic acid (LA), were found to be altered in the watermelon fruits silenced for specific DEGs, and these alterations were found to affect watermelon resistance to CGMMV infection. We consider that the findings presented in this paper provide not only new insights into the role of boron in watermelon resistance to CGMMV but also new knowledge for the establishment of an efficient and environmentally friendly control method for WBFD in watermelon fruits and possibly other cucurbit crops.

## 2 RESULTS

### 2.1 Foliar application of exogenous boron solution can alleviate CGMMV infection in watermelon fruits

To test the effect of boron application on watermelon resistance to CGMMV infection, we performed four treatments (i.e., phosphate‐buffered saline (P) + water (H), P + boron (B), CGMMV (C) + H, and C + B) on watermelon plants (Figure 1a). At 15 days after pollination (DAP), no obvious disease symptoms were observed in the watermelon fruits from all the assayed plants (Figure 1b). By 30 DAP, the fruits from the P + H‐, P + B‐ or C + B‐treated plants turned pink inside, while the fruits from the C + H‐treated plants showed some dark red areas near seeds (Figure 1b). By 45 DAP, the fruits from the P + H‐ or P + B‐treated plants showed uniformed red flesh inside, but the fruits from the C + H‐treated plants were smaller and had nonuniform dirty‐red flesh with some sponge‐like tissues near seeds. The fruits from the C + B‐treated plants had red flesh with much less or no sponge‐like tissues near seeds (Figure 1b), indicating that foliar application of exogenous boron solution reduced the CGMMV‐induced WBFD.

Northern blot and western blot assays showed that the accumulations of CGMMV genomic RNA and coat protein (CP) were increased significantly in the fruits from the C + H‐ or C + B‐treated plants as plant growth progressed. Compared to the fruits from the C + H‐treated plants, the accumulation level of CGMMV genomic RNA in the fruits from the C + B‐treated plants was decreased by 70% (15 DAP), 57% (30 DAP) and 22% (45 DAP). Consistent with this trend, viral CP accumulation in C + B vs. C + H comparisons decreased by 60%, 50% and 20% at 15, 30 and 45 DAP, respectively (Figure 1c). To further investigate the effect of exogenous boron application on CGMMV infection, we measured the firmness, fresh weight, and endogenous boron content of watermelon fruits. The results showed that fruit firmness and fresh weight decreased after CGMMV infection, but these two traits were partly or fully restored after the foliar application of exogenous boron solution (Figure 1d,e). The content of boron in the fruits from the P + B‐ or C + B‐treated plants was much higher than that in the fruits from the P + H‐ or C + H‐treated plants. It is noteworthy that the content of endogenous boron in the fruits from the C + H‐treated plants was significantly decreased compared to that in the fruits from the rest treatment plants, and this difference gradually increased as the plant growth progressed (Figure 1f). These findings indicate that CGMMV infection reduces boron content in fruits, while foliar application of exogenous boron solution can increase boron content and alleviate CGMMV infection.

### 2.2 CGMMV infection‐induced global transcriptomic changes in watermelon fruits

To investigate the effect of exogenous boron application on CGMMV infection, we analysed watermelon fruits from the P + H‐, P + B‐, C + H‐, and C + B‐treated plants at 15, 30, and 45 DAP through RNA‐Seq. In this study, each treatment had three biological replicates and a total of 36 fruit cDNA libraries were analysed (Table S1). After removal of low‐quality reads, a total of 231.44 GB clean reads were obtained with a Q30 percentage >93.08% and a GC content between 43.95% and 44.85% (Table S1). The clean reads were then mapped to the watermelon reference genome using HISAT2 software and resulted in 92.99%–95.94% mapped reads and 90.60%–94.08% unique mapped reads (Table S2). The clean reads data were deposited in the NCBI database (BioProject PRJNA694461). The relative expression levels of individual genes found in each sample were normalized using the FPKM values as the box plots (Figure S1).

### 2.3 Analysis of DEGs in different groups

**FIGURE 2:**
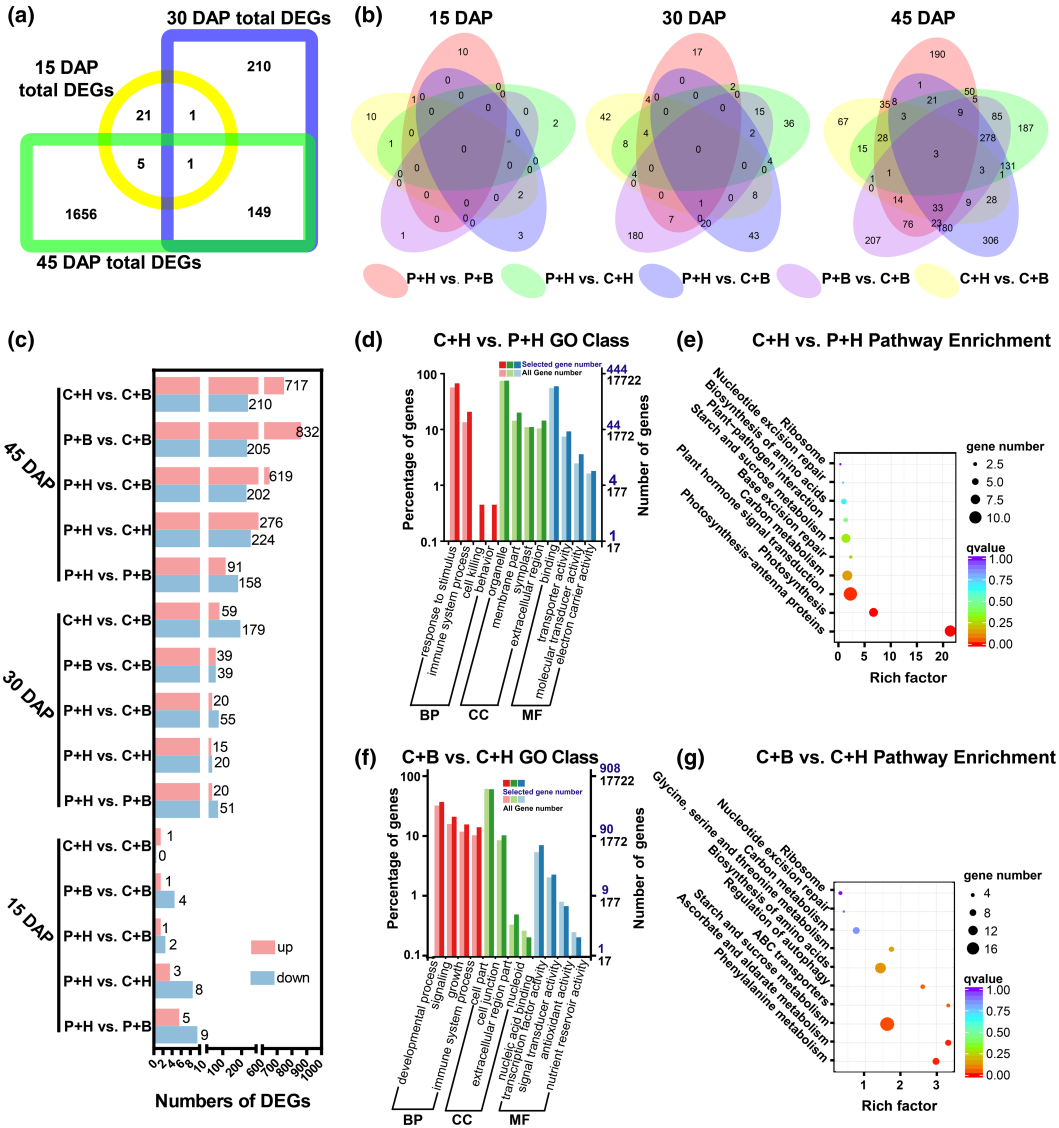
Statistical and enrichment analyses of differentially expressed genes (DEGs). (a, b) Venn diagrams showing total DEGs and the DEGs in five different comparisons (i.e., phosphate‐buffered saline [P] + water [H] vs. P + boron [B], P + H vs. CGMMV [C] + H, P + H vs. C + B, P + B vs. C + B, and C + H vs. C + B comparison) at 15, 30, and 45 days after pollination (DAP). (c) Statistical analyses of up‐ and down‐regulated DEGs in five different comparisons. (d, e) Gene ontology (GO) and KEGG analyses of DEGs observed in the fruits from the P + H‐ and C + H‐treated plants at three different sampling stages. (f, g) GO and KEGG analyses of DEGs in the fruits from the C + H‐ and C + B‐treated plants at three different sampling stages.

To investigate the temporal changes of gene expression induced by CGMMV infection in watermelon fruits, we conducted five different pairwise comparisons (i.e., P + B vs. P + H, C + H vs. P + H, C + B vs. P + H, C + B vs. P + B, and C + B vs. C + H) at 15, 30, and 45 DAP. Through these comparisons, a total of 28 DEGs were found among the comparisons at 15 DAP, 361 DEGs at 30 DAP, and 1811 DEGs at 45 DAP (Figure 2a,b). The results presented in Figure 2c show the numbers of up‐ or down‐regulated genes in each comparison. The results also show that more DEGs were in the C + B vs. P + H, C + B vs. P + B, or C + B vs. C + H comparisons than that in the P + B vs. P + H comparison at 45 DAP. In addition, more DEGs were in treatment at 45 DAP than that in the same treatment at 15 and 30 DAP.

To further investigate the response in watermelon fruits to CGMMV infection alone and exogenous boron during CGMMV infection, we analysed the DEGs found in the C + H vs. P + H or C + B vs. C + H comparisons at 15, 30, and 45 DAP using the GO terms and the KEGG pathway enrichment. A total of 444 DEGs were found in the C + H vs. P + H comparison and were annotated to GO terms under the categories response to stimulus, immune system process, and cell killing (Figure 2d and Table S3). The KEGG pathway analysis result indicated that the DEGs found in the C + H vs. P + H comparison were mainly in the categories photosynthesis, carbon metabolism, plant hormone signal transduction, and base excision repair (Figure 2e and Table S4). A total of 908 DEGs were found in the C + B vs. C + H comparison and were mainly assigned to the GO terms under the categories nucleic acid binding, transcription factor activity, antioxidant activity, and nutrient reservoir activity (Figure 2f and Table S3). The KEGG pathway analysis result indicated that the DEGs found in the C + B vs. C + H comparison were mainly in the categories phenylalanine metabolism, ascorbate and aldarate metabolism, and biosynthesis of amino acids (Figure 2g and Table S4). These findings indicate that CGMMV infection can significantly affect the physiological and biochemical responses in watermelon fruits, and foliar application of boron can induce host defence responses as well as antioxidation, transcription factor expression, and nutrition redistribution.

### 2.4 DEG coexpression network analysis

**FIGURE 3:**
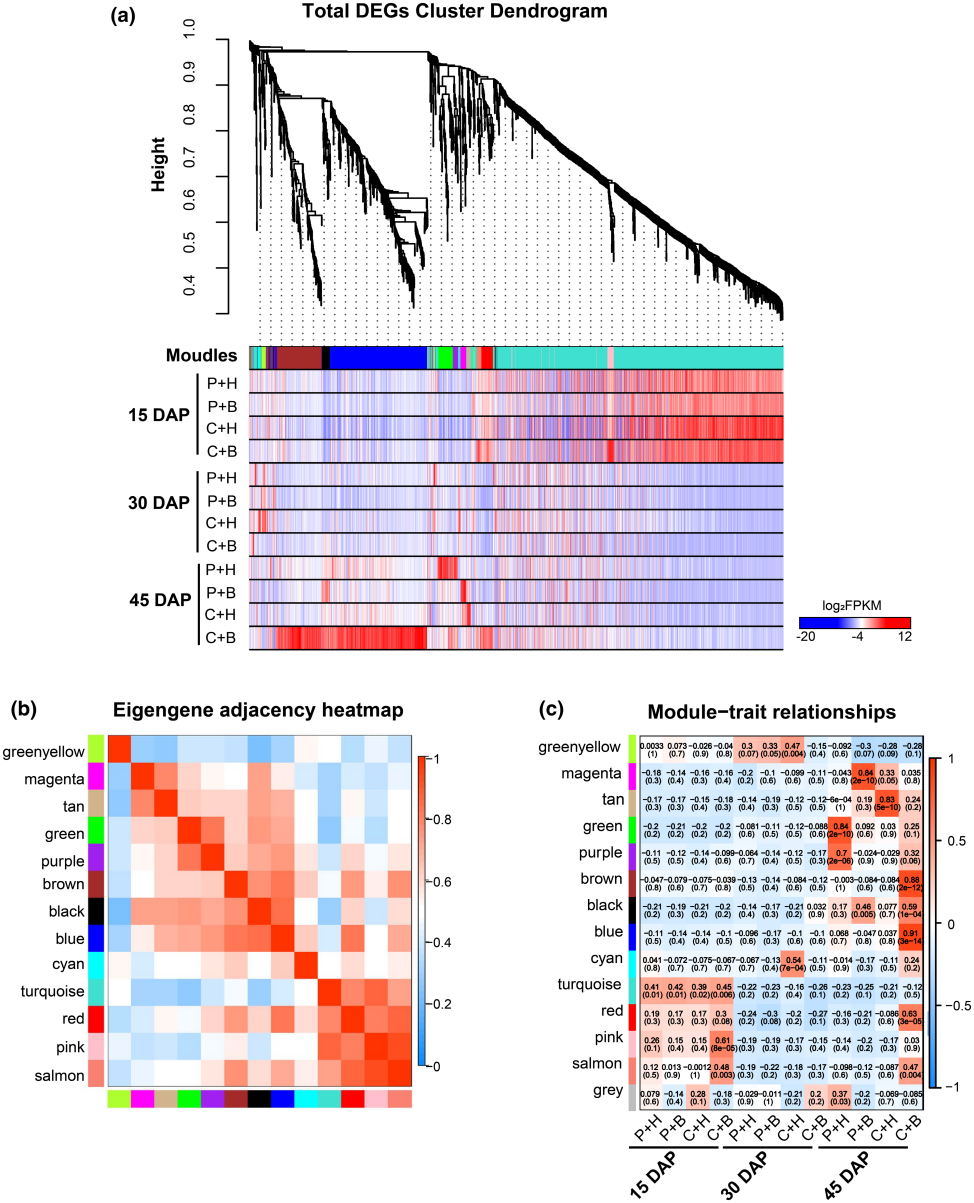
Weighted correlation network analysis (WGCNA) of differentially expressed genes (DEGs) in the fruits from the phosphate‐buffered saline (P) + water (H)‐, P + boron (B)‐, CGMMV (C) + H‐, and C + B‐treated plants at 15, 30, and 45 days after pollination (DAP). (a) Hierarchical cluster tree and heatmap of all DEGs. The hierarchical cluster tree shows coexpression modules identified through WGCNA. Each leaf in the tree represents one DEG. The major tree branches constitute 13 modules labelled with different colours. The heatmap shows the relative expressions of the whole DEGs in different modules. (b) Eigengene adjacency heatmap of the 13 modules shows the correlations among different modules, the darker red represents a higher correlation. (c) Associations between modules and traits. The colours of the modules are the same as that shown in (a) and (b). Each column represents each treatment at 15, 30, and 45 DAP. The numbers in individual cells represent the correlations and the *p* values.

To investigate the changes of gene regulatory networks in watermelon fruits, we analysed 2043 DEGs using WGCNA and identified 13 distinct gene regulatory network modules containing clusters of highly correlated genes based on pairwise correlation analysis result and gene expression trends. The numbers of DEGs in these modules ranged from 9 to 547 (Figure 3a and Table S5). The correlations among the 13 different modules are presented as a module eigengene adjacency heatmap (Figure 3b), and the relationships between the modules and treatments are shown in Figure 3c. The results shown in Figure 3b,c represent the blue module with 279 DEGs and the brown module with 130 DEGs in the same gene regulatory network. These DEGs were highly expressed in the fruits from the C + B‐treated plants at 45 DAP (Figure 3b,c), and were mainly involved in signal transduction, plant disease resistance, cell wall and plasma membrane, energy metabolism, and secondary metabolism, according to the topGO and KEGG analyses (Tables S6 and S7). Also in the blue and brown modules, several calmodulin‐like genes (e.g., *CML21*, *CML23*, *CML27*, *CML30*, *CML41*, and *CML48*) and serine/threonine protein kinase‐associated genes (e.g., *OXI1*, *PBS1*, *BIK1*, and *CIPK*) are known to be involved in signal transduction and immune responses; several transcription factors (TFs), including *MYB*s, *WRKY*s, and *NAC*s, are known to associate with plant disease resistance; *PE/PEI7* and *XTH23* are known to regulate cell wall modification; *SWEET*s can regulate sugar homeostasis; and *4CL*s are key enzymes involved in secondary metabolism and plant stress resistance (Table S5). The pink and red modules were highly correlated with the C + B treatment at 15 and 45 DAP, respectively. Most DEGs found in these two modules are associated with plant hormone and ion channels, including *IAA13* and *NIP5‐1* (Tables [Link], [Link], [Link]). The results of this study also showed that the DEGs in the tan module were up‐regulated in the fruits from the C + H‐treated plants at 45 DAP. These DEGs are known to associate with endomembrane system, cytochrome, and plant–pathogen interaction, and include *Cyt‐P450* and *RBohC* (Figure 3c, and Tables [Link], [Link], [Link]). The DEGs in the green and purple modules were significantly correlated in the fruits from the P + H‐treated plants at 45 DAP, and contained *ripening‐related expansin* involved in the developmental growth, and *HSP20.3* and *HSP22.9* in the protein processing in endoplasmic reticulum (Figure 3c, and Tables [Link], [Link], [Link]). The magenta module was significantly correlated with the P + B treatment at 45 DAP, and the DEGs in this module were enriched mainly in the lignin metabolic process and phenylpropanoid biosynthesis (Figure 3c, Tables S6 and S7).

Combination of global WGCNA, topGO, and KEGG analysis results revealed that most of the DEGs identified in this study are involved in plant hormone, signal transport, cell wall, plasma membrane, energy metabolism, and secondary metabolism. In particular, the DEGs in the blue and brown modules are associated with watermelon responses to the C + B treatment.

### 2.5 Analyses of functions of DEGs involved in polyamine and phytohormone production

**FIGURE 4:**
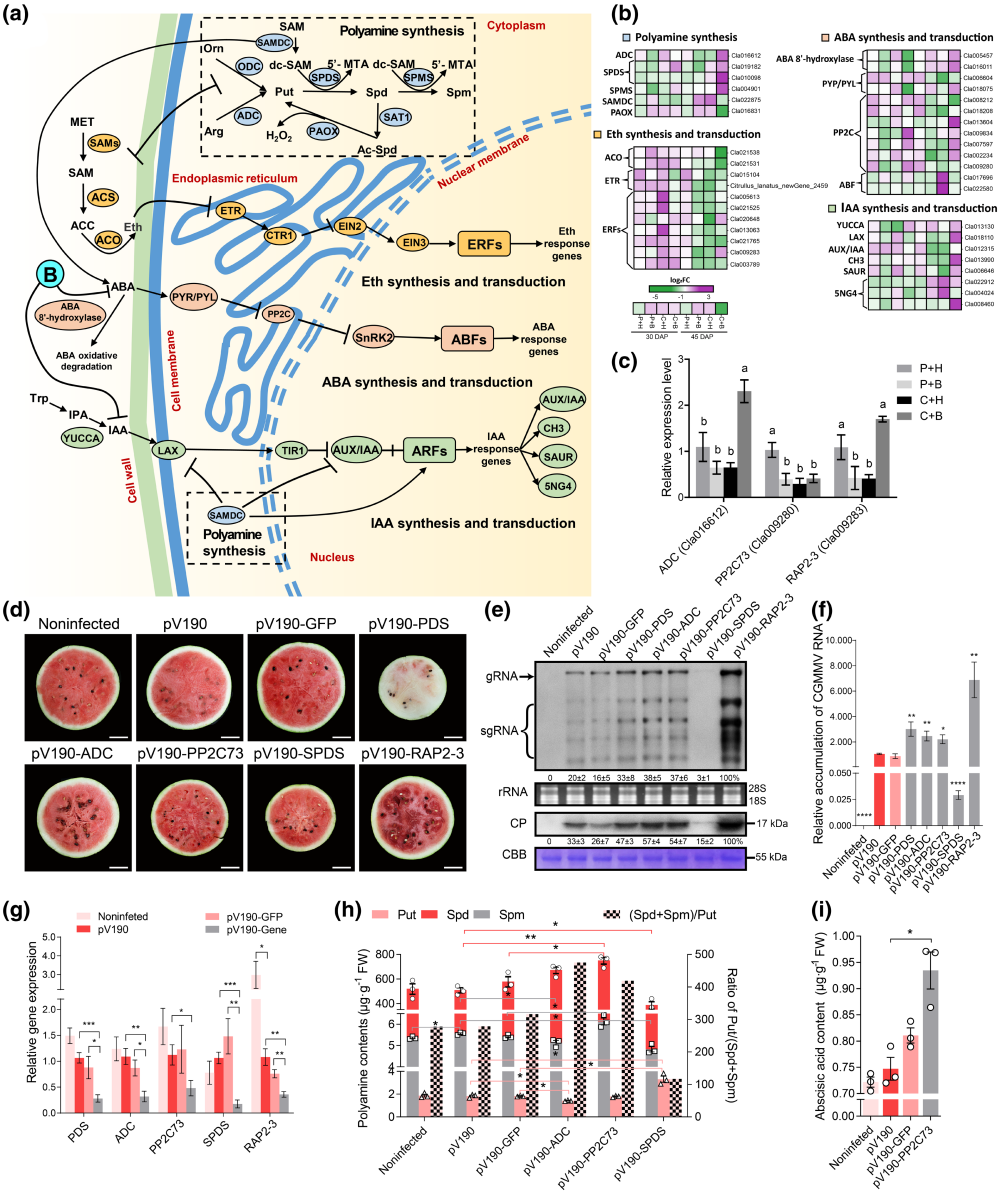
Effects of the crosstalk between polyamine biosynthesis and phytohormone signalling on CGMMV infection. (a) Diagram describing the crosstalk between polyamine biosynthesis and phytohormone signalling. (b) Expression patterns of differentially expressed genes (DEGs) associated with polyamine biosynthesis and phytohormone signalling. The heatmap shows the expressions of DEGs associated with polyamines, ethylene (Eth), abscisic acid (ABA), and auxin (IAA) biosynthesis, and signal transductions at 30 and 45 days after pollination (DAP). (c) Validation of RNA‐Seq results through reverse transcription‐quantitative PCR (RT‐qPCR) (*n* = 9). P, phosphate‐buffered saline; H, water; B, boron; C, CGMMV. (d) Watermelon blood flesh disease symptoms in watermelon fruits from various gene‐silenced watermelon plants or controls at 45 days postinoculation (dpi). (e, f) Determination of CGMMV accumulation in the fruits from various gene‐silenced watermelon plants or controls at 45 dpi by northern blot, western blot, and RT‐qPCR analyses. CP, coat protein; CBB, Coomassie brilliant blue. (g) Silencing efficiencies of individual genes through virus‐induced gene silencing (VIGS) were determined through RT‐qPCR (*n* = 9). (h, i) Analyses of polyamines and ABA contents in the fruits from various gene‐silenced watermelon plants or controls. The results are expressed as the mean ± *SD*. The statistical significances were determined using one‐way analysis of variance followed by Duncan's multiple comparison test (*p* < 0.05). Other results were determined using the two‐tailed *t* test. *p < 0.05, **p < 0.01, ***p < 0.001. Scale bar = 5 cm. Abbreviations of genes and compounds are explained in Table S10.

Putrescine (Put), spermidine (Spd), and spermine (Spm) are polyamines that are known to participate in plant antiviral response, fruit ripening, and hormone regulation (Anwar, 2015; Fernandez‐Calvino et al., 2014; Guo et al., 2018). Six DEGs identified at 45 DAP were found to associate with Put, Spd, and Spm biosynthesis, as well as mutual transformation of polyamines. In the C + B vs. C + H comparison, the expressions of arginine decarboxylase (*ADC*) and spermidine synthases (*SPDS*s and *SPMS*) were found to be up‐regulated, while the expression of polyamine oxidase (*PAOX*) was down‐regulated. Also, the expression of S‐adenosylmethionine decarboxylase (*SAMDC*) was significantly up‐regulated in the C + H vs. P + H comparison, but down‐regulated in the C + B vs. C + H comparison (Figure 4a,b and Table S8). The 11 ethylene pathway‐associated DEGs were ethylene synthases (*ACO*s), ethylene receptors (*ETR*s), and ethylene‐responsive transcription factors (*ERF*s). Of these *ERF*s, most were found to be significantly down‐regulated in the C + H vs. P + H comparison. Two *ACO*s were found to be significantly down‐regulated, while several *ERF*s, including *RAP2‐3* (Cla009283), were found to be significantly up‐regulated in the C + B vs. C + H comparison (Figure 4a,b and Table S8). Moreover, 13 DEGs were found to be related to catabolic processes and abscisic acid (ABA) signal transduction. In the C + B vs. C + H comparison, two ABA 8′‐hydroxylase genes were found to be significantly up‐regulated. Among the protein phosphatase 2Cs (*PP2C*s), the expression of *PP2C73* (a negative regulator of ABA signalling) was significantly down‐regulated in the C + H vs. P + H comparison (Figure 4a,b and Table S8). Eight DEGs were found to be involved in the synthesis, signalling, and downstream elements of auxin (IAA), and among these DEGs the expression of auxin biosynthesis rate‐limiting enzyme (*YUCCA*), auxin transporter (*LAX*), auxin‐responsive protein (*AUX/IAA*), and downstream elements (i.e., *CH3*, *SAUR*) were significantly up‐regulated in the C + B vs. C + H comparison (Figure 4a,b and Table S8).

To validate the above findings, we analysed the expression of three selected DEGs through reverse transcription‐quantitative PCR (RT‐qPCR). The results showed that the expressions of *ADC* and *RAP2‐3* were indeed significantly up‐regulated in the C + B vs. C + H comparison, while the expression of *PP2C73* was significantly down‐regulated in the C + H vs. P + H comparison, consistent with the RNA‐Seq result obtained at 45 DAP (Figure 4b,c and Table S8). To investigate the functions of the polyamine or phytohormone pathway‐associated DEGs on WBFD symptom development, we performed VIGS experiments using a CGMMV‐based VIGS vector. By 45 days postagroinfiltration (dpi), the fruits from the pV190‐PDS‐inoculated plants showed not only photobleaching symptoms in leaves, but also white pulp in fruits (Figures 4d and S2a,b), demonstrating a good silencing effect by this VIGS vector in watermelon plants and fruits. At the same time, silencing of *RAP2‐3* expression caused more severe WBFD symptoms, and silencing of *ADC* or *PP2C73* expression also aggravated WBFD symptoms through VIGS compared to that in fruits infected with pV190 or pV190‐GFP. In contrast, silencing of *SPDS* expression prevented visible WBFD symptoms in watermelon fruits compared to the fruits infected with pV190 or pV190‐GFP (Figure 4d). Results of northern blot, western blot, and RT‐qPCR assays showed that the accumulation level of CGMMV in watermelon fruits was correlated with the severity of WBFD symptoms (Figure 4d–f). The RT‐qPCR results showed that the gene silencing efficiencies obtained in this study ranged from 52% to 82% (Figure 4g). To determine the effects of *ADC*, *PP2C73*, and *SPDS* on polyamine and plant hormone biogenesis, we first silenced these genes individually through VIGS and then measured the contents of Put, Spd, Spm, and ABA in the fruits. The results revealed that the content of Put was significantly reduced, leading to an increased (Spd + Spm):Put ratio in fruits from the *ADC*‐silenced plants. In addition, an increased (Spd + Spm):Put ratio was found in the fruits from the *PP2C73*‐silenced plants accompanied by a much higher ABA content. In contrast, silencing of *SPDS* expression resulted in a low ratio of (Spd + Spm):Put and a higher Put content in the fruits (Figure 4h,i).

The above results indicated that (a) polyamine and IAA biogenesis, and ABA catabolism were promoted after the C + B treatment, while ethylene biosynthesis was inhibited; (b) *SPDS* is required for efficient CGMMV accumulation, while *RAP2‐3* plays a role in watermelon resistance to CGMMV infection; (c) the ratio of (Spd + Spm):Put is correlated with the relative severity of WBFD in fruits; and (d) the content of Put may have a negative impact on CGMMV infection.

### 2.6 Roles of DEGs in watermelon antiviral defence signalling

**FIGURE 5:**
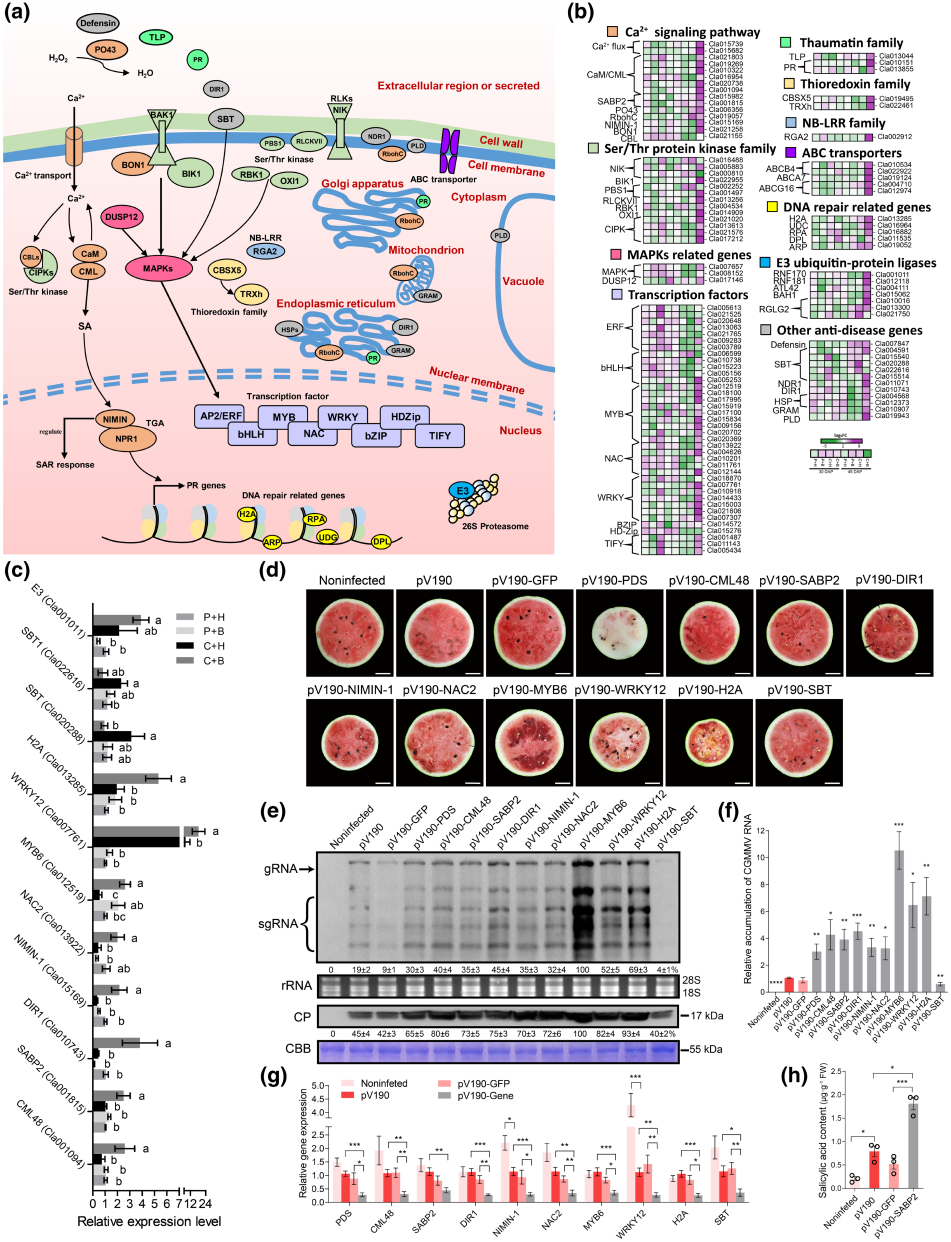
Boron‐mediated multiple defence‐related pathways during CGMMV infection. (a) Representation of antiviral defence signalling. (b) Expression patterns of immune signal transduction‐ and disease resistance‐associated differentially expressed genes (DEGs) in watermelon fruits. Heatmaps describe the expressions of DEGs associated with calmodulin, Ser/Thr protein kinase family, MAPKs, transcription factors, thaumatin family, thioredoxin family, NB‐LRR family, ABC transporters, DNA repair pathway, E3 ubiquitin‐protein ligases, and other disease resistance genes at 30 and 45 days after pollination (DAP). (c) Validations of RNA‐Seq results through reverse transcription‐quantitative PCR (RT‐qPCR) (*n* = 9). P, phosphate‐buffered saline; H, water; B, boron; C, CGMMV. (d) Watermelon blood flesh disease symptoms in the fruits from various gene‐silenced watermelon plants collected at 45 days postinoculation (dpi). (e, f) Determination of CGMMV accumulation in the fruits from various gene‐silenced watermelon plants or controls at 45 dpi by northern blot, western blot, and RT‐qPCR analyses. CP, coat protein; CBB, Coomassie brilliant blue. (g) RT‐qPCR analysis of virus‐induced gene silencing (VIGS)‐induced target gene silencing efficiencies (*n* = 9). (h) Salicylic acid (SA) content in the *SABP2*‐silenced watermelon fruits or controls (*n* = 3). Results are presented as the mean ± *SD*. The statistical significance between the treatments was determined using one‐way analysis of variance followed by Duncan's multiple comparison test (*p* < 0.05). Other results were analysed using the two‐tailed *t* test. *p < 0.05, **p < 0.01, ***p < 0.001. Scale bar = 5 cm. Abbreviations of genes and compounds are explained in Table S10.

Plants have evolved many defence strategies to resist virus replication and movement (Calil & Fontes, 2017). In this study, we found that most DEGs identified in the C + H vs. P + H or the C + B vs. C + H comparison were involved in the Ca^2+^ signalling pathway (i.e., *CaM/CML*, *SABP2*, *PO43*, *NIMIN‐1*, and *BON1*), Ser/Thr protein kinase family (i.e., *RLCKVII*, *RBK1*, *OXI1*, and *CIPKs*), MAPKs, *TF*s (i.e., *MYB*s, *NAC*s, *WRKY*s, and *TIFY*s), thaumatin family (i.e., *TLP* and *PR*s), thioredoxin family (i.e., *CBSX5* and *TRXh*), NB‐LRR family (*RGA2*), ABC transporters, DNA repair‐related genes (i.e., *H2A*, *UDC*, *RPA*, and *DPL*), and E3 ubiquitin‐protein ligases (*UbE3*s). These DEGs were up‐regulated in the C + B vs. C + H comparison, while *ERF*s and *NAC*s were down‐regulated in the C + H vs. P + H comparison at 45 DAP. The expressions of *MYB6* and *MYB305* were found to be up‐regulated in the C + B vs. C + H comparison at 30 and 45 DAP. In the C + B vs. C + H comparison, the expressions of *CIPK6*, *H2A*, and *lipid‐transfer protein DIR1* were down‐regulated at 30 DAP and then up‐regulated at 45 DAP. At 45 DAP, the expressions of *Subtilisin‐like proteases* (*SBT* and *SBT1*) were found to be up‐regulated in the C + H vs. P + H comparison and down‐regulated in the C + B vs. C + H comparison (Figure 5a,b and Table S8). A comparison analysis of the expressions of these genes through RT‐qPCR agreed with most of the RNA‐Seq results (Figure 5b,c and Table S8).

To further validate the above findings, we silenced the expression of *CML48*, *SABP2*, *DIR1*, *NIMIN‐1*, *NAC2*, *MYB6*, *WRKY12*, *H2A*, and *SBT* in watermelons through VIGS. The fruits collected from the *SBT*‐silenced plants showed very mild WBFD symptoms and accumulated much less CGMMV. In contrast, the fruits collected from the *MYB6*‐, *WRKY12*‐ or *H2A*‐silenced plants showed more severe WBFD symptoms and accumulated more CGMMV (Figure 5d–f). In silenced plants, these target genes were down‐regulated to 19%–40% of their levels (Figure 5g). In this study, we also found that the content of salicylic acid (SA) in the fruits from the *SABP2*‐silenced plants was significantly higher than that in the fruits from the pV190‐ or pV190‐GFP‐inoculated control plants (Figure 5h).

The above results proved that (a) the Ca^2+^ signalling pathway, immune signalling transduction, TF regulation, DNA repair systems, ubiquitin pathway, and broad‐spectrum resistance were activated by exogenous boron under CGMMV infection, and (b) *SBT* was required for efficient CGMMV accumulation, while *MYB6*, *WRKY12*, and *H2A* participated in watermelon resistance to CGMMV infection.

### 2.7 Roles of DEGs in cell wall and plasma membrane functions, and energy and secondary metabolism

**FIGURE 6:**
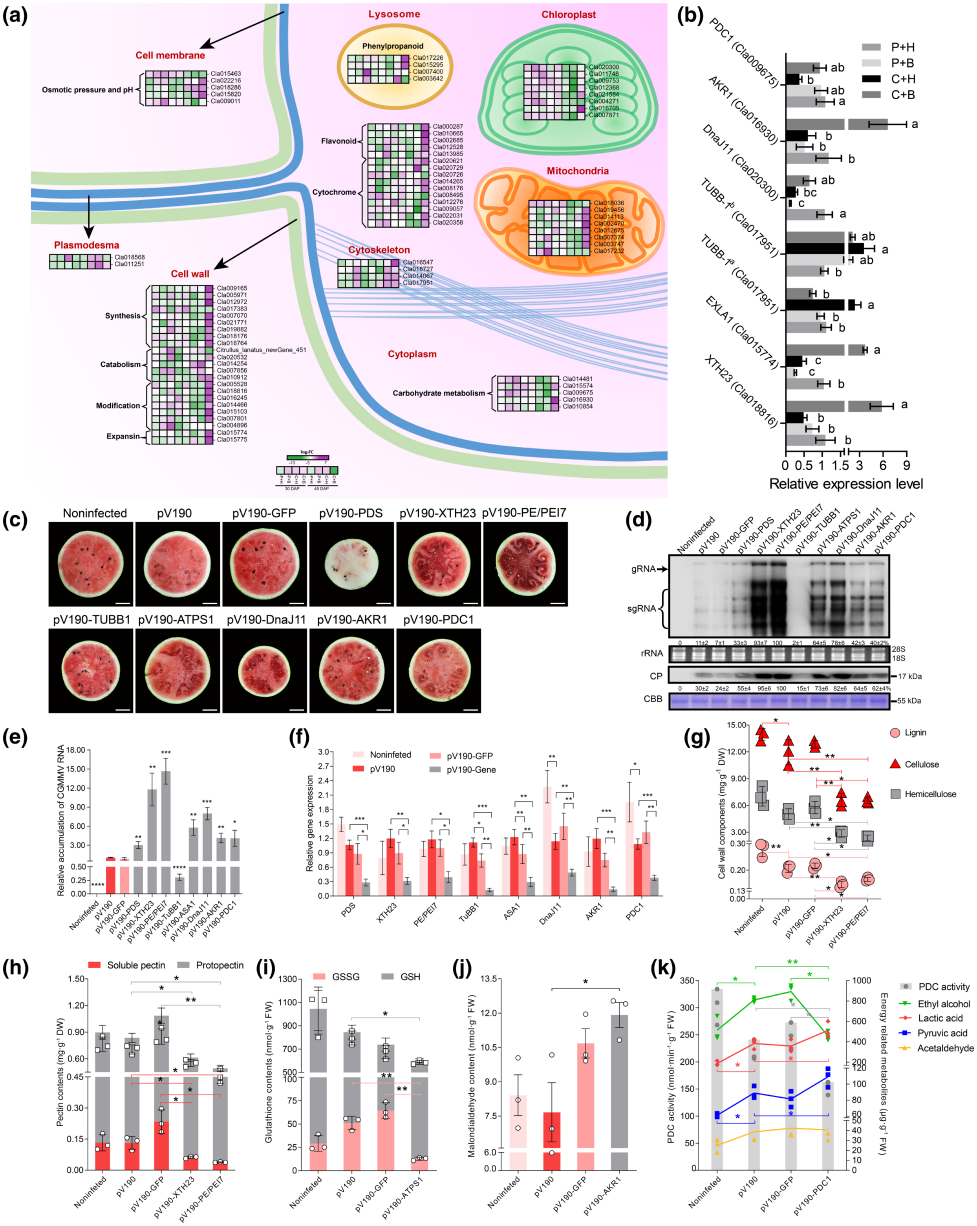
Effects of cell wall modification‐, energy metabolism‐ and cytoskeleton‐associated differentially expressed genes (DEGs) on CGMMV infection. (a) Subcellular localizations and expression patterns of DEGs associated with cell wall, cell membrane, energy, and secondary metabolism in cells. The heatmap shows the expression patterns of DEGs associated with cell wall, cell membrane, chloroplast, cytoskeleton, lysosome, mitochondria, and plasmodesmata at 30 and 45 days after pollination (DAP). (b) Reverse transcription‐quantitative PCR (RT‐qPCR) validation of selected DEGs identified through RNA‐Seq (*n* = 9). *TUBB1* ^*a*^ is a DEG found at 30 DAP, while *TUBB1* ^*b*^ is a DEG found at 45 DAP. P, phosphate‐buffered saline; H, water; B, boron; C, CGMMV. (c) Watermelon blood flesh disease symptoms in the fruits from the gene‐silenced watermelon plants or controls at 45 days postinoculation (dpi). (d, e) Determination of CGMMV accumulation in the fruits from various gene‐silenced watermelon plants or controls at 45 dpi by northern blot, western blot and RT‐qPCR analyses. CP, coat protein; CBB, Coomassie brilliant blue. (f) RT‐qPCR analyses of virus‐induced gene silencing efficiencies (*n* = 9). (g–k) Analyses of contents of lignin, cellulose, hemicellulose, soluble pectin, protopectin, oxidized glutathione (GSSG), reduced glutathione (GSH), malondialdehyde, pyruvic acid, acetaldehyde, ethanol, lactic acid, and pyruvate decarboxylase (PDC) enzyme activity in various gene‐silenced watermelon plants or controls (*n* = 3).

Boron plays important roles in cell wall biosynthesis, and cytoskeleton and membrane functions (Camacho‐Cristóbal, Rexach, et al., 2008). In this study, we identified 34 cell wall and plasma membrane‐associated DEGs in the C + H vs. P + H and C + B vs. C + H comparisons at 30 and 45 DAP (Figure 6a and Table S8). Most DEGs found in the C + B vs. C + H comparison at 45 DAP were up‐regulated and are known to be involved in cell wall biosynthesis (*API*, *CSLD3*, *CESA4*, *COBRA*, *GRP*, and *CCR1*) or cell wall modification (*XTH1*, *XTH23*, *XTH30*, *PE/PEI*, *PE/PEI7 PE/PEI51*, and *EXLA1*), while several cell wall catabolism‐associated DEGs (*BG12*, *Pel13*, *β‐gal3*, and *CenA*) were found to be down‐regulated at 30 and 45 DAP. In addition, at 30 DAP, the expressions of *RTNLB21*, *TUB*, and *TUBB1* were significantly down‐regulated in the C + B vs. C + H comparison, while the expression of *TUBB1* was up‐regulated in the C + H vs. P + H comparison at 45 DAP. In this study, several identified DEGs (*TIP*s, *PIP*s, and *TDT*) are known to be associated with water permeability and pH homeostasis.

The expression of 40 energy and secondary metabolism‐associated genes was found to be affected by the C + B treatment (Figure 6a and Table S8). At 45 DAP, the expression patterns of chloroplast‐associated DEGs (*CAB*s, *PSI‐E A*, *DnaJ11*, *ATPS1*, and *TL17*) were similar to that of carbohydrate metabolism‐associated DEGs (*PDC1*, *AKR*s, *TPPJ*, *BFRUCT1*, and *CWINV1*). All of these DEGs were found to be up‐regulated in the C + B vs. C + H comparison and down‐regulated in the C + H vs. P + H comparison. In addition, most mitochondria‐associated DEGs (*AGXT2*, *UCP5*, *AOX2*, *CACL*, *FIS1*, and *TIM23*) were only up‐regulated in the C + B vs. C + H comparison, and their expression patterns were similar to that of secondary metabolism‐associated DEGs, including the DEGs associated with phenylpropanoid (*4CL*s), flavonoid (*CHS2*, *F3H*, *IFR*, and *F3'H*), and cytochrome (*CYP81D1*, *CYP84A1*, *CYP86A1*, *CYP734A1*, *CYB5*, and *CYB561D*).

To validate the expression of these RNA‐Seq results, we analysed six DEGs identified in the 30 and 45 DAP samples through RT‐qPCR. The results confirmed that the expressions of *XTH23*, *EXLA1*, *DnaJ11*, *AKR1*, and *PDC1* were up‐regulated in the C + B vs. C + H comparison at 45 DAP, while the expression of *TUBB1* was up‐regulated in the C + H vs. P + H comparison at 30 and 45 DAP (Figure 6a,b and Table S8). We then analysed the correlations between seven DEGs (*XTH23*, *PE/PEI7*, *TUBB1*, *ATPS1*, *DnaJ11*, *AKR1*, and *PDC1*) and WBFD through VIGS. The results showed that silencing of *XTH23*, *PE/PEI7*, *ATPS1*, *DnaJ11*, *AKR1* or *PDC1* expression caused stronger WBFD symptoms in watermelon fruits and higher virus accumulation compared to that in the fruits infected with pV190 or pV190‐GFP (Figure 6c–e). When the expression of *TUBB1* was silenced through VIGS, the fruits showed no obvious WBFD symptoms and no virus accumulation (Figure 6c–e). The silencing efficiency of each target gene was determined to be 52%–88% through RT‐qPCR (Figure 6f).

To investigate the effect of CGMMV infection on the biosynthesis of cell‐wall polysaccharides, sulphur‐containing compounds, cytotoxic compounds, and carbon metabolites, we measured the contents of lignin, cellulose, hemicellulose, soluble pectin, and protopectin in the fruits from the *XTH23*‐ and *PE/PEI7*‐silenced plants, the contents of GSSG and GSH in the fruits from the *ATPS1*‐silenced plants, the MDA content in the fruits from the *AKR1*‐silenced plants, and the contents of pyruvic acid (PA), acetaldehyde (AA), ethanol (EtOH), and LA as well as the activity of pyruvate decarboxylase (PDC) in the fruits from the *PDC1*‐silenced plants. The results showed that silencing of *XTH23* or *PE/PEI7* expression in plants significantly decreased the contents of lignin, celluloses, and pectin (Figure 6g,h), and silencing of *ATPS1* expression decreased the contents of GSSG, GSH, and GSSG + GSH (Figure 6i,j). In addition, silencing of *PDC1* expression reduced the PDC activity and EtOH content, but increased the contents of PA and LA (Figure 6k).

### 2.8 The effects of individual silencing homologues of watermelon genes on CGMMV infection in *Nicotiana benthamiana*

To further explore the roles of watermelon genes in resistance to CGMMV infection, we silenced 12 homologues of the above watermelon genes using the TRV‐based VIGS system in *N. benthamiana* plants (Table S9). At 19 dpi, silencing *NbPDS* in *N. benthamiana* plants caused drastic photobleaching symptoms in leaves, and the gene silencing efficiencies obtained in this assay ranged from 48% to 90% (Figure S3a,b). Among these, *NbRAP2‐3*‐, *NbMYB48*‐, *NbWRKY11*‐, *NbXTH28*‐silenced plants showed severe mosaic symptoms in upper leaves with massive CGMMV accumulation (Figure S3a,c), and *NbADC*‐, *NbCML36*‐, *NbNAC2*‐, *NbH2A*‐, *NbATPS1*‐silenced plants also showed mosaic symptoms and accumulated more CGMMV in young leaves (Figure S3a,c). Moreover, silencing *NbSPDS* and *NbTUB* caused no mosaic symptoms and less CGMMV accumulation in upper leaves compared with those in TRV‐GFP‐treated plants (Figure S3a,c). These results in *N. benthamiana* were basically consistent with those of pV190 VIGS assays in watermelon, supporting the conclusion of the resistance functions of these watermelon genes to CGMMV infection.

## 3 DISCUSSION

CGMMV infection‐induced WBFD is a devastating disease in watermelon production. In this study, we analysed the function of boron in CGMMV infection in watermelon fruits. Our results show that application of exogenous boron can efficiently inhibit CGMMV infection and minimize WBFD symptoms in watermelon fruits. Moreover, we have identified through RNA‐Seq multiple genes whose expression was regulated by exogenous boron application and/or CGMMV infection. Most of the DEGs identified in this study were found in the 45 DAP samples, suggesting that watermelon resistance to CGMMV infection in the later stage is more complicated. To validate this speculation, we selected 20 DEGs and analysed their roles in watermelon resistance to CGMMV infection using a CGMMV‐based VIGS vector reported previously (Liu et al., 2020). The results of pV190 VIGS assays for watermelon leaf were consistent with those for fruit except for *DnaJ11* (Figures S2, 4 and 5). In addition, the results of TRV VIGS assays showed that 12 homologues in *N. benthamiana* played the same antiviral roles as the homologous genes in watermelon (Figure S3), which further confirmed the reliability of pV190 in functional verification of watermelon fruit genes in resistance to CGMMV infection. We consider that this VIGS procedure can provide researchers with a useful tool for reverse genetics to study gene functions in watermelon and possibly many other cucurbit plants.

### 3.1 Crosstalk between polyamines and phytohormones can promote or restrict CGMMV infection

Polyamines are essential for plant growth and development and stress responses (Anwar, 2015), including responses to virus infection (Olga et al., 2018). In 2019, Olga and colleagues reported that ADC is a key enzyme involved in Put biosynthesis, and Spd and Spm are synthesized from Put through catalysis by SPDS and SPMS, respectively. In this study, we have found that compared with CGMMV infection alone, boron application significantly up‐regulated the expression of *ADC* in the CGMMV‐infected watermelon fruits (Figure 4a,b and Table S8). In contrast, the expression of SAMDC, a rate‐limiting enzyme that catalyses S‐adenosylmethionine (SAM) for Spd and Spm production (Guo et al., 2018), was induced after CGMMV infection and then suppressed after application of exogenous boron (Figure 4a,b and Table S8). These findings indicate that CGMMV infection induces Spd and Spm production in watermelon fruits. However, after exogenous boron application, watermelon starts to synthesize more Put. This finding supports earlier reports that impairment of Put biosynthesis increases TRV infection in *Arabidopsis* plants (Fernandez‐Calvino et al., 2014), and binding of Spd and Spm to DNA and RNA are required during virus replication (Olga et al., 2018). Moreover, a greater ratio of (Spd + Spm):Put is known to promote fruit ripening and softening, and accelerate ABA biosynthesis (Guo et al., 2018), and to alter the expression of PP2C proteins and to regulate ABA production (Anwar, 2015). In this study, we found that more CGMMV accumulated in fruits from the *ADC*‐silenced plants, which had a higher (Spd + Spm):Put ratio and a lower Put content, and in the *PP2C73*‐silenced plants, which had a higher (Spd + Spm):Put ratio and a higher ABA content. In contrast, less CGMMV was detected in the *SPDS*‐silenced plants, which had a lower (Spd + Spm):Put ratio and a higher Put content. Based on the above findings, we propose that knockdown of *ADC* expression suppresses Put production, and knockdown of *PP2C73* expression increases Spd and Spm production, both of which lead to an aggravated virus infection. In addition, a higher (Spd + Spm):Put ratio and a higher ABA content can promote fruit senescence, leading to stronger WBFD symptoms in watermelon fruits. Because silencing of *SPDS* expression in watermelon plants resulted in a relatively higher Put content in fruits, these fruits accumulated much less CGMMV and showed much weaker WBFD symptoms (Figure 4h,i).

In the crosstalk between polyamines and phytohormones (Figure 4a), down‐regulation of *SAMDC* expression can inhibit ABA production while promoting IAA production (Guo et al., 2018). Because SAM serves as a common precursor, there may be an equilibrium between polyamines and ethylene synthesis (Anwar, 2015; Guo et al., 2018). Moreover, boron deficiency and sufficiency can increase and decrease IAA and ABA content, respectively (Eggert & Von Wirén, 2017). In this study, the expression of ethylene‐associated DEGs was found to be down‐regulated, while the IAA biosynthesis‐associated or ABA catabolism‐associated DEGs were found to be up‐regulated after C + B treatment (Figure 4a,b and Table S8). We have also found that the boron content was significantly increased in the watermelon fruits from the C + B‐treated plants compared to that in the fruits from the C + H‐treated plants (Figure 1f), suggesting that application of exogenous boron can alter ABA, IAA, and ethylene biosynthesis as well as polyamine pathways under CGMMV stress in watermelon fruits. Downstream phytohormones are known to involve in plant disease resistance. For example, ERFs have been shown to participate in plant resistance to virus infections, including TMV (Fischer & Dröge‐Laser, 2004). In this study, after silencing *RAP2‐3* expression, CGMMV accumulation and WBFD symptoms were enhanced (Figure 4d–f), indicating that *RAP2‐3* is a positive regulator of watermelon resistance to CGMMV infection. Because the expression of *RAP2‐3* was suppressed in the C + H‐treated plants, we consider that suppression of *RAP2‐3* expression is one of the strategies CGMMV used to counteract host resistance to ensure its infection in plant (Figure 4a,b and Table S8). In contrast, the expression of *RAP2‐3* was significantly up‐regulated in the C + B‐treated plants (Figure 4a,b and Table S8), indicating that application of exogenous boron can up‐regulate *RAP2‐3* expression to alleviate CGMMV infection and WBFD symptom development.

### 3.2 Effect of exogenous boron on other antiviral defence responses

After recognition of pathogens, plants activate various signalling pathways to induce defence responses (Ramirez‐Prado et al., 2018). It was reported that calmodulin (CaM), calmodulin‐like proteins (CMLs), and calcineurin B‐like proteins (CBLs) can convert Ca^2+^ signals to induce plant immune responses to bacterial, fungal, and virus invasion (Aldon et al., 2018). CaM is also known to induce SA signalling (Jeon et al., 2017) and SABP2 (an SA receptor) is required for resistance to TMV (Kumar & Klessig, 2003). NIMIN is regulated by SA and can interact with SA sensor protein NPR1 to promote pathogenesis‐related protein (PR) gene expression and systemic acquired resistance (SAR) (Figure 5a) (Hermann et al., 2013). In this study, we have found that the expressions of *CaM‐1*, *CML*s, *SABP2*, and *NIMIN‐1* were up‐regulated in the fruits from the C + B treated plants (Figure 5a,b). Our VIGS assay results showed that silencing of *CML48*, *SABP2*, and *NIMIN‐1* expression enhanced CGMMV accumulation, and the SA content in the *SABP2*‐silenced fruits was significantly increased (Figure 5d–f). Therefore, we consider that application of exogenous boron can activate Ca^2+^ signalling and initiate SAR in watermelon plant. We also consider *CML48*, *SABP2* and *NIMIN‐1* to be positive regulators of watermelon resistance to CGMMV infection.

Sophisticated antiviral innate immunity systems of plants, that is, PAMP‐triggered immunity (*BAK1*, *BIK1*) (Calil & Fontes, 2017; Ramirez‐Prado et al., 2018), effector‐triggered immunity (*PBS1*, *NB‐LRR*) (Calil & Fontes, 2017; Lee & Kim, 2015), and NIK1‐mediated signalling (Calil & Fontes, 2017; Machado et al., 2015), can activate intricate defence signalling cascades, including the activations of TFs by MAPKs to resist pathogen infections (Ramirez‐Prado et al., 2018) (Figure 5a). In this study, we have determined that the expressions of *BIK1*, *NIKs*, *PBS1*, *RGA2*, and *MAPKKKA*s were altered by exogenous boron in response to CGMMV infection in the fruits (Figure 5a,b and Table S8). These altered gene expression patterns may also activate host antiviral signalling. Furthermore, the expression of several TFs, especially *NAC*s, *MYB*s, and *WRKY*s, were found to be affected by CGMMV infection alone and exogenous boron under CGMMV infection (Figure 5a,b and Table S8). *NAC* can interact with *WRKY*, *MYB*, and *TGA* to regulate the response to tomato yellow leaf curl virus infection (Huang et al., 2017). In this study, we have found that silencing of *NAC2*, *MYB6*, and *WRKY12* expression enhanced CGMMV infection, and silencing of *MYB6* expression also caused more severe WBFD symptoms. In contrast, the expression of these *TF*s were up‐regulated in the fruits from the C + B‐treated plants, suggesting that application of exogenous boron can increase *NAC2*, *MYB6*, and *WRKY12* expression to alleviate CGMMV infection and WBFD symptom development (Figure 5a–f and Table S8).

Numerous RNA viruses cause DNA damage in host cells during their lifecycles (Ryan et al., 2016), H2A is a key component in histone octamers and is responsible for DNA replication and repair (Zhou et al., 2015). Lipid transfer proteins (LTPs) are required for SAR long‐distance signalling to resist pepper mosaic mottle virus infection (Sarowar et al., 2009). In our study, the expression of *H2A* and *LTP‐DIR1* was down‐regulated at 30 DAP and then up‐regulated at 45 DAP in the C + B vs. C + H comparison (Figure 5a,b and Table S8). In our VIGS assays, we have found that silencing of *H2A* or *DIR1* expression enhanced CGMMV infection, and silencing of *H2A* expression also reduced watermelon fruit size (Figures 5d–f and S6), indicating that boron application can affect *H2A* and *DIR1* expression to regulate CGMMV infection. Another gene, *SBT*, is known to function in pathogen recognition, immune priming, and defence response (Figueiredo et al., 2014). In this study, the expression of *SBT* was up‐regulated in the CGMMV‐infected fruits, but down‐regulated in the fruits from the C + B‐treated plants (Figure 5a,b). Silencing of *SBT* expression through VIGS suppressed CGMMV infection in watermelon fruits but had no effect on fruit weight and quality (Figures 5d–f and S6), suggesting that CGMMV can hijack SBT to ensure its successful infection. Because silencing of *SBT* expression had no clear effect on fruit weight and quality, we consider *SBT* to be a candidate in watermelon resistance breeding.

### 3.3 Cell wall modification‐ and energy metabolism‐associated DEGs enhance host resistance while cytoskeleton‐associated DEGs benefit virus infection

The cell wall is a primary barrier to a variety of phytopathogens; viruses, bacteria, and fungi must penetrate it to start the infection process (Smirnova & Kochetov, 2016). Cell wall modification is a crucial element in host defence against biotic stresses (Miedes & Lorences, 2007; Tenhaken, 2015). Xyloglucan endotransglucosylase/hydrolase (XTH) is a hemicellulosic repairing enzyme that can remodel cell walls (Miedes & Lorences, 2007, Tenhaken, 2015). XTH participates in rice resistance to rice stripe virus infection (Zheng et al., 2013). PE/PEI is a cell wall modification enzyme and has two domains (i.e., pectin methylesterase [PME] and pectin methylesterase inhibitor [PMEI]). The PME domain is known to catalyse the demethylation of pectin and to interact with the viral movement protein, while the PMEI domain is known to counteract the actions of plant PMEs to limit virus spread (Lionetti et al., 2014). In this study, the expression of *XTH23* and *PE/PEI7* was up‐regulated in the fruits from the C + B treated plants (Figure 6a and Table S8). Compared with the fruits from the control plants, the fruits from the *XTH23‐* or *PE/PEI7*‐silenced plants showed much stronger WBFD symptoms and accumulated more CGMMV, but these fruits had much less lignin, cellulose, hemicellulose, soluble pectin, and protopectin (Figure 6c–e,g–k), suggesting that boron application can induce *XTH23* and *PE/PEI7* expression to strengthen cell walls and to enhance the PMEI function, respectively. In contrast, silencing of *XTH23* and *PE/PEI7* expression reduced the contents of celluloses, pectins, and lignin, the essential cell wall components (Meents et al., 2018; Verbančič et al., 2018), which destroyed the cell wall barrier, helped viral spread, and aggravated WBFD symptoms.

The cytoskeleton and tubulin are required for virus transport (McLean et al., 1995); boron deprivation can increase the tubulin content in *Arabidopsis* (Camacho‐Cristóbal, Rexach, et al., 2008). In our study, we found that CGMMV infection in watermelon fruits was associated with low boron content (Figure 1f). We also found that at 45 DAP, the expression of *TUBB1* was up‐regulated in the C + H vs. P + H comparison and down‐regulated at 30 DAP in the C + B vs. C + H comparison, indicating that *TUBB1* was induced by CGMMV infection and inhibited by exogenous boron (Figure 6a and Table S8). Moreover, the fruits from the *TUBB1*‐silenced plants showed significantly reduced CGMMV accumulation and no WBFD symptoms (Figure 6c–e). These findings agree with previous reports and indicate that *TUBB1* has a role in CGMMV infection in watermelon.

In this study, the expression of chloroplast *DnaJ11* and *ATPS1* was found to be up‐regulated in the C + B vs. C + H comparison and down‐regulated in the C + H vs. P + H comparison at 45 DAP (Figure 6a and Table S8). DnaJ proteins are cochaperones that can interact with HSP70s to control protein homeostasis (Bolhassani & Agi, 2019). HSP70s are also known to act as host defence factors or virus help factors to accelerate virus replication and spread in plants (Hýsková et al., 2021). Our VIGS assay result showed that silencing of *DnaJ11* expression exacerbated CGMMV infection and WBFD symptom development in watermelon fruits (Figure 6c–e). We speculate that the up‐regulation of *DnaJ11* expression through boron application may increase HSP70 activities to enhance watermelon antiviral resistance. ATPS is a key enzyme involved in sulphur assimilation and biosynthesis of GSH and homo‐GSH (h‐GSH), which are crucial in host stress tolerance (Anjum et al., 2015). In our study, we found that silencing of *ATPS1* expression through VIGS increased CGMMV infection and reduced GSSG, GSH, and GSSG + GSH contents in watermelon fruits (Figure 6c–e,i), suggesting that ATPS1 can alleviate WBFD symptoms in watermelon fruits through regulation of sulphur‐compound synthesis.

AKR1 has been shown to improve tobacco and rice seed longevity through reducing the contents of cytotoxic compounds (Nisarga et al., 2017). In our study, the expression of *AKR1* and *AKR2* was found to be up‐regulated by exogenous boron under CGMMV infection, while silencing of *AKR1* expression increased CGMMV infection and MDA content (Figure 6a–e,j). Pyruvate is a glycolytic product and is catalysed by PDC to produce acetaldehyde and then ethanol, not LA, in plants, leading to less damage and less acidification in watermelon fruits (Papastolopoulou et al., 2018). We also found that the expression of *PDC1* was significantly down‐regulated in the C + H vs. P + H comparison and up‐regulated in the C + B vs. C + H comparison in the late fruit growth stage, and silencing of *PDC1* expression through VIGS enhanced CGMMV infection, increased pyruvic acid and LA contents, but reduced ethanol content in watermelon fruits (Figure 6a–e,k). Therefore, we consider that application of exogenous boron can induce *AKR1* and *PDC1* expression to reduce MDA content and increase ethanol production, but not LA, leading to an attenuated cell acidification in watermelon fruits.

**FIGURE 7:**
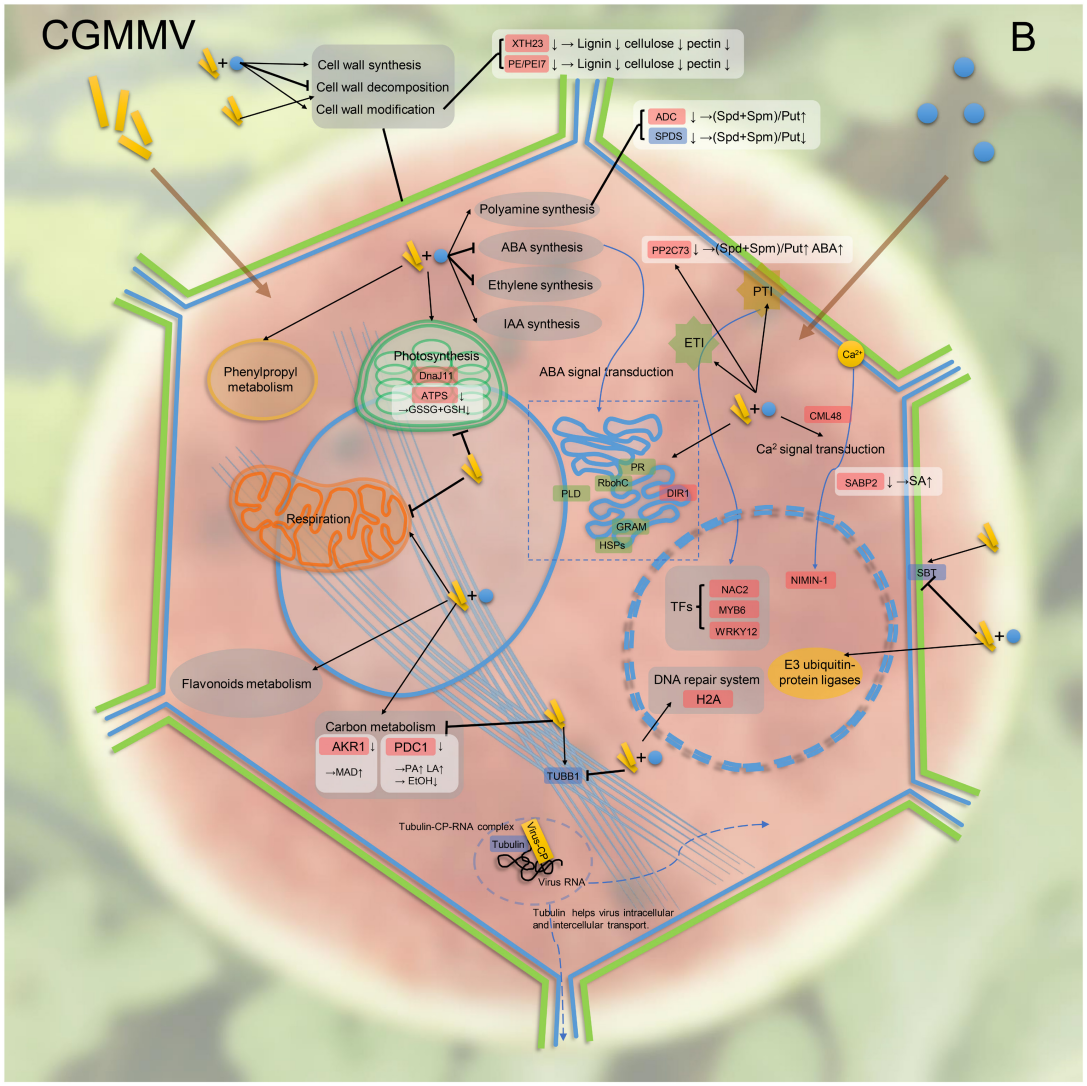
A working model for boron‐mediated watermelon resistance to CGMMV infection. Genes in pink boxes represent watermelon genes for resistance to CGMMV infection, verified through virus‐induced gene silencing (VIGS) assays. Genes in blue boxes represent watermelon genes for susceptibility to CGMMV infection, verified through VIGS assays. The white boxes show the expression changes of differentially expressed genes (DEGs) related to metabolite biosynthesis in each gene‐silenced plant. Genes in green boxes represent DEGs in the cytomembrane and are up‐regulated by exogenous boron under viral infection in the fruits. The biological processes shown in the grey boxes represent the major pathways regulated by both virus infection and exogenous boron.

In summary, application of exogenous boron can significantly inhibit CGMMV infection and alleviate WBFD symptoms in watermelon fruits. In multiple resistance‐related pathways, most DEGs involved in polyamine and IAA synthesis, ABA catabolism, defence‐related pathways, cell wall modification, energy, and secondary metabolism were induced, while those related to ethylene synthesis, cell wall catabolism, and plasma membrane function were inhibited by exogenous boron under CGMMV infection. Among these DEGs, *SPDS*, *SBT*, and *TUBB1* are required for CGMMV infection and WBFD symptom development, while *RAP2‐3*, *MYB6*, *WRKY12*, *H2A*, *XTH23*, *PE/PEI7*, *DnaJ11*, and *ATPS1* are involved in watermelon resistance to CGMMV infection. Furthermore, higher Put content contributes to an antiviral effect, while a higher (Spd + Spm):Put ratio and lower lignin, celluloses, pectins, and GSHs contents aggravate WBFD symptom development in fruits. In addition, higher MDA and LA content accelerates watermelon fruit decay and acidification (Figure 7). Taken together, we consider that the results presented in this paper provide new knowledge on the boron‐mediated watermelon resistance to CGMMV infection. This new knowledge can benefit the establishment of effective and environmentally friendly control methods for CGMMV infection in watermelon fields.

## 4 EXPERIMENTAL PROCEDURES

### 4.1 Plant growth

Watermelon cv. Qilin plants were cultivated in a greenhouse in the Xinmin region (42°00′32.44″ N, 122°47′51.02″ E; Liaoning province, China). The greenhouse was maintained at approximately 30 and 19°C (day and night), 40%–60% air humidity, and an average 12 h (light) and 12 h (dark) photoperiod. During the experiment, watermelon plants were drip‐irrigated with water and a potassium sulphate solution. Watermelon vine pruning, plot arrangement, plot protection line arrangement, and insect protection net installation were done according to local field management practice. The same growth conditions were also used for watermelon plants for fruit VIGS assays. In addition, watermelon seedlings used for leaf VIGS assays were grown in pots inside a growth chamber maintained at 25°C and a 16 h light and 8 h dark photoperiod.

### 4.2 Virus inoculation and exogenous boron treatment

CGMMV (GenBank ID: KY040049) was maintained in bottle gourd plants inside the laboratory. Watermelon plants were sprayed with water or a boron solution before and after inoculation with crude extracts from CGMMV‐infected leaf tissues or phosphate‐buffered saline (PBS, mock inoculation) as described in Figure 1a. For the treatments of spraying watermelon leaves, a low concentration (30 mg/L H_3_BO_3_) boron solution was used at 10 and 20 days after transplantation, and a high concentration (300 mg/L H_3_BO_3_) boron solution was used at 30 and 41 days after transplant. In this study, watermelon plants were treated with PBS and water (P + H), PBS and boron solution (P + B), CGMMV and water (C + H), or CGMMV and boron solution (C + B). Each treatment had three biological replicates and each replicate had at least nine plants. At 15, 30, and 45 DAP, a total of 36 fruits were harvested from the assayed watermelon plants and analysed for fruit weight, firmness, boron content, virus accumulation, and gene expression.

### 4.3 Global gene expression analysis

Total RNA was isolated from the collected 36 fruit samples using an RNAprep Pure Plant Kit (Tiangen). mRNA was then isolated from individual total RNA samples (1 μg each) using the AMPure XP system (Beckman Coulter) and then fragmented into about 240 bp fragments. Reverse‐transcribed cDNA fragments were prepared, purified, end repaired, and then ligated to adapters using the Next Ultra RNA Library Prep kit as instructed (New England Biolabs). The final cDNA libraries were constructed through enrichment PCR followed by sequencing on the Illumina HiSeq 4000 sequencing platform by Biomarker Biology Technology Co. Ltd (Beijing, China).

Clean reads were obtained by removing reads containing adapter from raw data and mapped to the watermelon reference genome database (http://www.cucurbitgenomics.org/organism/1) using HISAT2 software (http://ccb.jhu.edu/software/hisat2/index.shtml). Relative expression levels of genes in the 36 libraries were calculated and then normalized based on the numbers of fragments per kilobase of transcript per million mapped reads (FPKM). DEGs in the libraries were identified using the false discovery rate (FDR) <0.05 and |log_2_ (fold change)| ≥1. Seven publicly available databases (Nr, Nt, Pfam, KOG/COG, Swiss‐Prot, KO, and GO) were then used for gene annotations to obtain the comprehensive information on the functions of Unigenes. GO and KEGG enrichment analyses were used to find the main biological functions and pathways of the DEGs found in different libraries.

### 4.4 Gene coexpression network analysis

A total of 2043 DEGs in Table S1 were analysed using the WGCNA v. 3.1.1 package in R (Langfelder & Horvath, 2008) to identify the coexpressed gene modules. A gene expression adjacency matrix was constructed to show the network topology with an unsigned type of topological overlap matrix (TOM), a power β of 5, a minModuleSize of 10, and a mergeCutHeight value of 0.25.

### 4.5 VIGS assay

Twenty genes involved in the polyamine and phytohormone pathways (*ADC*, *PP2C73*, *SPDS*, and *RAP2‐3*), immune resistance (*CML4*8, *SABP2*, *DIR1*, *NIMIN‐1*, *NAC2*, *MYB6*, *WRKY12*, *H2A*, and *SBT*), cell wall and cell membrane function (*XTH23*, *PE/PEI7*, and *TUBB1*), and energy and secondary metabolism (*ATPS1*, *DnaJ11*, *AKR1*, and *PDC1*) were analysed for their functions during CGMMV infection in watermelon plants using a CGMMV‐based VIGS vector (pV190) (Liu et al., 2020). Briefly, approximately 260 bp fragments representing the partial sequence of one of the 20 selected genes were PCR amplified using the gene‐specific primers (Table S9). The resulting PCR products were individually cloned into the pV190 vector using the ClonExpress II One Step Cloning Kit (Vazyme). The constructed plasmids were individually transformed into *Agrobacterium tumefaciens* GV3101. *Agrobacterium* cultures were individually prepared as described (Liu et al., 2020) and diluted to OD_600_ = 1.0. The fully expanded cotyledons and the four lowest true leaves of individual 12‐leaf‐stage watermelon plants (about 1 week before fruit setting) were infiltrated with an *Agrobacterium* culture containing a specific VIGS vector. The infiltrated plants were grown inside a growth chamber or in a greenhouse for further analyses as described (Liu et al., 2020). Plants infiltrated with an *Agrobacterium* culture carrying the empty pV190 VIGS vector or a pV190‐GFP vector carrying a 266 bp fragment representing the partial sequence of a *GFP* gene or a pV190‐PDS vector carrying a 300 bp fragment representing the partial sequence of watermelon *PDS* gene were used as controls. Leaf tissues and fruits were collected from these assayed plants at 32 and at 45 dpi, respectively, and were analysed for gene silencing efficiency and CGMMV accumulation.

Twelve genes in *N. benthamiana* that were homologous with the above watermelon genes were selected to be silenced by TRV‐based VIGS vector (Jiao et al., 2020). These approximately 330 bp fragments representing the partial sequence of the 12 genes were amplified individually from an *N. benthamiana* cDNA through PCR using the PrimeSTAR Max DNA polymerase (TaKaRa) and specific primers (Table S9). After digestion with *Bam*HI and *Xho*I restriction enzymes (TaKaRa), the resulting PCR fragments were cloned into the pTRV2 plasmid. These new plasmids and pTRV1 plasmid were transformed individually into *A. tumefaciens* GV3101. After propagation, *Agrobacterium* culture harbouring pTRV1 was mixed with an equal amount of *Agrobacterium* culture harbouring new plasmids and diluted to OD_600_ = 0.5. These mixed cultures were infiltrated into leaves of 9–10‐leaf stage *N*. *benthamiana* plants. Then, the upper noninfiltrated *N*. *benthamiana* leaves were mechanically inoculated with CGMMV crude extracts at 10 dpi. Plants infiltrated with an *Agrobacterium* culture carrying pTRV2 with a 311 bp fragment representing the partial sequence of a *GFP* gene were used as control. Monitoring of plant symptoms and sampling of leaf samples were performed at 9 dpi with CGMMV, and these samples were used to measure gene silencing efficiency and CGMMV accumulation.

### 4.6 RT‐qPCR

Total RNA was isolated as described above and used for cDNA synthesis using the HiScript III RT SuperMix supplemented with a gDNA wiper (Vazyme). Relative expression levels of the assayed genes and CGMMV RNAs were determined through RT‐qPCR using the ChamQ SYBR qPCR Master Mix (Vazyme) on the StepOnePlus Real‐Time PCR System (Applied Biosystems). The relative expression level of watermelon *Actin* gene (Cla007792) was used as an internal control. The RT‐qPCR results were calculated using the 2^−ΔΔ*C*t^ method (Schefe et al., 2006). The primers used in this study are listed in Table S9. Three biological replicates with three technique replicates each were used for each gene.

### 4.7 Northern blot and western blot analysis

For northern blot assay, total RNA was isolated from mixed watermelon samples. Each mixed sample represented the leaves or fruits from nine watermelon plants, and the RNA samples were separated in 1.5% denaturing agarose gels containing 5% formaldehyde. The separated RNAs were transferred to Hybond‐N^+^ nylon membranes (Amersham) through capillary action and the membranes were probed with a digoxigenin‐labelled RNA probe, synthesized using a pair of CGMMV‐specific primers (CGMMV‐F and CGMMV‐R) using the DIG Northern Starter Kit as instructed (Roche) (Table S9). The labelling signal was visualized using the 5200 chemical luminous imaging system (Tanon).

For western blot assay, total protein was extracted from the samples described above using the Plant Protein Extraction Kit (Solarbio), quantified using the bicinchoninic acid (BCA) Protein Assay Kit (TaKaRa), and then separated by 12% SDS‐PAGE. The separated proteins were transferred to 0.20 μm polyvinylidene fluoride (PVDF) membranes (Millipore). The resulting membranes were blocked with a bovine serum albumin solution, and then incubated in a 1/4000 diluted anti‐CGMMV coat protein monoclonal antibody solution as described (Shang et al., 2011), followed by a 1/5000 diluted goat anti‐mouse IgG alkaline phosphatase (AP)‐conjugated secondary antibody (Sigma‐Aldrich). The detection signal was visualized by addition of the chemiluminescent substrate CDP‐star (Roche) and then on a 5200 chemical luminous imaging system (Tanon).

### 4.8 Physiological index measurements

Boron contents in the fruits collected from the four treatments were measured through inductively coupled plasma‐mass spectrometry (ICP‐MS, Agilent 7500a; Agilent Technologies) as described previously (Moustafa‐Farag et al., 2016). Free ABA in the pV190‐PP2C73‐infected fruits and free SA in the pV190‐SABP2‐infected fruits were measured through high‐performance liquid chromatography‐mass spectrometry (HPLC‐MS) at a wavelength of 254 nm in a mobile phase (55:45 vol/vol methanol/0.1% acetic acid solution) and at 302 nm in a mobile phase (35:65 vol/vol methanol/0.1% acetic acid solution), respectively, with specific modifications (Yu et al., 2020). The contents of polyamines (i.e., Put, Spd, and Spm) in the pV190‐ADC‐, pV190‐PP2C73‐, and pV190‐SPDS‐infected fruits were first measured at a wavelength of 254 nm in a mobile phase (60:40 vol/vol 90% acetonitrile + 10% [0.1% acetic acid containing 0.1 M acetic amine]/10% acetonitrile + 90% [0.1% acetic acid containing 0.1 M acetic amine]) as described (Oliveira et al., 2020). The calibration curves of ABA, SA, Put, Spd, and Spm standards (Sigma‐Aldrich) on the HPLC system (Shimadzu Corp.) were then used to calculate the contents of the above compounds. The MDA content in the V190‐AKR1‐infected fruits was measured at a wavelength of 532 nm using a method reported previously (Moustafa‐Farag et al., 2016). The lignin, cellulose, hemicellulose, soluble pectin, and protopectin contents in the pV190‐XTH23‐ and pV190‐PE/PEI7‐infected fruits were measured using Physiological Assay Kits as instructed (Suzhou Grace Biotechnology Co.) on the basis of dry fruit weight. The PDC activity and the contents of pyruvic acid, acetaldehyde, ethanol, and LA in the pV190‐PDC1‐infected fruits were also measured using the Physiological Assay Kits as instructed (Suzhou Grace Biotechnology Co.) on the basis of fresh fruit weight. The plants treated by pV190 and pV190‐GFP, and noninfected plants acted as controls. Each assayed sample represented fruits from nine plants and each measurement had three biological replicates.

### 4.9 Statistical analysis

The RT‐qPCR and biological index measurement results were further analysed using one‐way analysis of variance followed by Duncan's multiple comparison test. The VIGS assay results were analysed using Student's two‐tailed *t* test. The statistical significances were determined using SPSS Statistics 25 (IBM Inc.).

## AUTHOR CONTRIBUTIONS

X.B., H.Y., X.L., L.Z., M.A., Z.X., and Y.W. conducted experiments. X.B., H.Y., X.L., and L.Z. provided boron foliar spraying and VIGS assay. X.B., H.Y., and X.L. performed transcriptomic analysis and curated data. Y.W., Z.X., and M.A. conceived the study, analysed data, and provided supervision. X.B. prepared figures. X.B., Z.X., and Y.W. wrote the article with contributions from all authors.

## CONFLICT OF INTEREST

The authors declare no conflict of interest.

## Supporting information


**FIGURE S1** FPKM values of differentially expressed gene (DEG) relative expression in each sample via box plots

**FIGURE S2** Leaf virus‐induced gene silencing (VIGS) assays in greenhouse and in growth chamber of polyamines and phytohormones. (a) White spot symptoms of watermelon peel at 32 days postinoculation (dpi) by pV190‐PDS treatment. (b) Mosaic symptoms of various gene‐silenced watermelon leaves or controls in the greenhouse at 32 dpi. (c) Northern blot and western blot analyses of the accumulation of CGMMV RNA and coat protein (CP) from various gene‐silenced watermelon leaves or controls in a greenhouse. (d) Silencing efficiencies of individual genes through VIGS were determined through reverse transcription‐quantitative PCR (RT‐qPCR) in watermelon leaves of greenhouse (*n* = 9). (e) Accumulation of CGMMV from various gene‐silenced watermelon leaves or controls in the greenhouse by RT‐qPCR (*n* = 9). (f) Mosaic symptoms of various gene‐silenced watermelon leaves or controls in a growth chamber at 32 dpi. (g) Northern blot and western blot analyses of the accumulation of CGMMV RNA and CP from various gene‐silenced watermelon leaves or controls in a growth chamber. (h) Growth vigour of VIGS‐treated and noninfected watermelon seedlings in a growth chamber at 32 dpi. (i) Silencing efficiencies of individual genes through VIGS were determined through RT‐qPCR in watermelon leaves of growth chamber (*n* = 9). (j) Accumulation of CGMMV from various gene‐silenced watermelon leaves or controls in the growth chamber by RT‐qPCR (*n* = 9). (k) Plant height of VIGS‐treated and noninfected watermelon seedlings in the growth chamber at 32 dpi (*n* = 12). The results were obtained at 32 dpi and expressed as the mean ± *SD*, using a two‐tailed *t*‐test (*p < 0.05, **p < 0.01, ***p < 0.001, ****p < 0.0001). Scale bar = 10 cm

**FIGURE S3** The effects of individual silencing 12 homologues of watermelon genes on CGMMV infection by TRV‐based virus‐induced gene silencing (VIGS) systems in *Nicotiana benthamiana*. (a) Mosaic symptoms of various gene‐silenced leaves in *N*. *benthamiana* or controls at 9 days postinoculation (dpi) with CGMMV. (b) Gene silencing efficiency of TRV VIGS assays were determined through reverse transcription‐quantitative PCR (RT‐qPCR) (*n* = 9). (c) CGMMV accumulation from various gene‐silenced leaves in *N*. *benthamiana* or controls at 9 dpi with CGMMV were determined through RT‐qPCR (*n* = 9). The results were expressed as the mean ± *SD*, using a two‐tailed *t* test (*p < 0.05, **p < 0.01, ***p < 0.001, ****p < 0.0001)

**FIGURE S4** Leaf virus‐induced gene silencing (VIGS) assays in greenhouse and in growth chamber of multiple defence pathways. (a) Mosaic symptoms of various gene‐silenced watermelon leaves or controls in the greenhouse at 32 days postinoculation (dpi). (b) Northern blot and western blot analyses of the accumulation of CGMMV RNA and coat protein (CP) from various gene‐silenced watermelon leaves or controls in a greenhouse. (c) Silencing efficiencies of individual genes through VIGS were determined through reverse transcription‐quantitative PCR (RT‐qPCR) in watermelon leaves of greenhouse (*n* = 9). (d) Accumulation of CGMMV from various gene‐silenced watermelon leaves or controls in the greenhouse by RT‐qPCR (*n* = 9). (e) Mosaic symptoms of various gene‐silenced watermelon leaves or controls in a growth chamber at 32 dpi. (f) Northern blot and western blot analyses of the accumulation of CGMMV RNA and CP from various gene‐silenced watermelon leaves or controls in a growth chamber. (g) Growth vigour of VIGS‐treated and noninfected watermelon seedlings in a growth chamber at 32 dpi. (h) Silencing efficiencies of individual genes through VIGS were determined through RT‐qPCR in watermelon leaves of growth chamber (*n* = 9). (i) Accumulation of CGMMV from various gene‐silenced watermelon leaves or controls in the growth chamber by RT‐qPCR (*n* = 9). (j) Plant height of VIGS‐treated and noninfected watermelon seedlings in the growth chamber at 32 dpi (*n* = 12). The results were obtained at 32 dpi and expressed as the mean ± *SD*, using a two‐tailed *t* test (*p < 0.05, **p < 0.01, ***p < 0.001, ****p < 0.0001). Scale bar = 10 cm

**FIGURE S5** Leaf virus‐induced gene silencing (VIGS) assays in greenhouse and in growth chamber of cell wall, plasmalemma, and energy and secondary metabolisms. (a) Mosaic symptoms of various gene‐silenced watermelon leaves or controls in the greenhouse at 32 days postinoculation (dpi). (b) Northern blot and western blot analyses of the accumulation of CGMMV RNA and coat protein (CP) from various gene‐silenced watermelon leaves or controls in a greenhouse. (c) Silencing efficiencies of individual genes through VIGS were determined through reverse transcription‐quantitative PCR (RT‐qPCR) in watermelon leaves of greenhouse (*n* = 9). (d) Accumulation of CGMMV from various gene‐silenced watermelon leaves or controls in the greenhouse by RT‐qPCR (*n* = 9). (e) Mosaic symptoms of various gene‐silenced watermelon leaves or controls in a growth chamber at 32 dpi. (f) Northern blot and western blot analyses of the accumulation of CGMMV RNA and CP from various gene‐silenced watermelon leaves or controls in a growth chamber. (g) Growth vigour of VIGS‐treated and noninfected watermelon seedlings in a growth chamber at 32 dpi. (h) Silencing efficiencies of individual genes through VIGS were determined through RT‐qPCR in watermelon leaves of growth chamber (*n* = 9). (i) Accumulation of CGMMV from various gene‐silenced watermelon leaves or controls in the growth chamber by RT‐qPCR (*n* = 9). (j) Plant height of VIGS‐treated and noninfected watermelon seedlings in the growth chamber at 32 dpi (*n* = 12). The results were obtained at 32 dpi and expressed as the mean ± *SD*, using a two‐tailed *t* test (*p < 0.05, **p < 0.01, ***p < 0.001, ****p < 0.0001). Scale bar = 10 cm

**FIGURE S6** Fruit diameter of gene‐silenced watermelons (*n* = 12). The results are expressed as the mean ± *SD*, using a two‐tailed *t* test (*p < 0.05, **p < 0.01, ***p < 0.001)

**TABLE S1** Summary of sequencing reads after filtering

**TABLE S2** Read numbers mapped onto the watermelon reference genome

**TABLE S3** Gene Ontology (GO) analysis of differentially expressed genes (DEGs) of three sampling stages (15, 30 and 45 days after pollination [DAP]) in CGMMV (C) + water (H) vs. phosphate‐buffered saline (P) + H and C + boron (B) vs. C + H

**TABLE S4** KEGG analysis of differentially expressed genes (DEGs) of three sampling stages (15, 30 and 45 days after pollination [DAP]) in CGMMV (C) + water (H) vs. phosphate‐buffered saline (P) + H and C + boron (B) vs. C + H

**TABLE S5** Total differentially expressed genes (DEGs) and DEGs in each module of weighted correlation network analysis

**TABLE S6** topGene Ontology (GO) analysis of differentially expressed genes (DEGs) of modules

**TABLE S7** KEGG analysis of differentially expressed genes (DEGs) of modules

**TABLE S8** Differentially expressed genes (DEGs) of various biological functional classifications

**TABLE S9** Specific primers used for quantitative PCR, virus‐induced gene silencing, and northern blot assays

**TABLE S10** Abbreviations of the genes and compounds in Figures 4 and 5

## Data Availability

RNA‐Seq data are available from the NCBI Gene Expression Omnibus at www.ncbi.nlm.nih.gov/bioproject with accession number PRJNA694461. All other data that support the findings of this study are available from the corresponding author upon reasonable request.
