# Supplementary figures and images for: A novel strategy for improving watermelon resistance to cucumber green mottle mosaic virus by exogenous boron application
Source: Mol Plant Pathol. 2022 Jun 7;23(9):1361–80. doi: 10.1111/mpp.13234 (PMC9366068; doi:10.1111/mpp.13234)

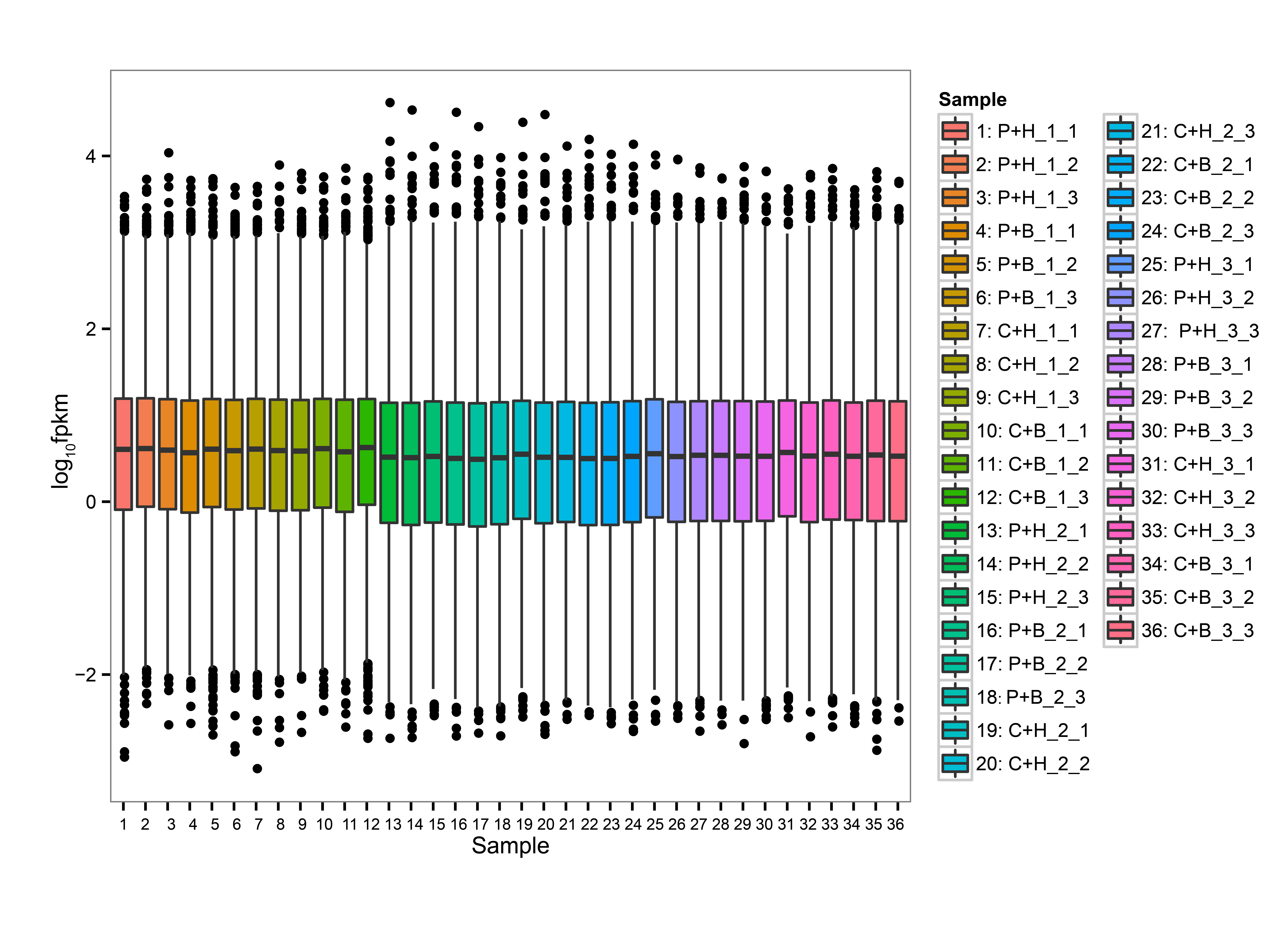

Supplement: Supplementary file 1 — FIGURE S1 FPKM values of differentially expressed gene (DEG) relative expression in each sample via box plots [file MPP-23-1361-s016.png]

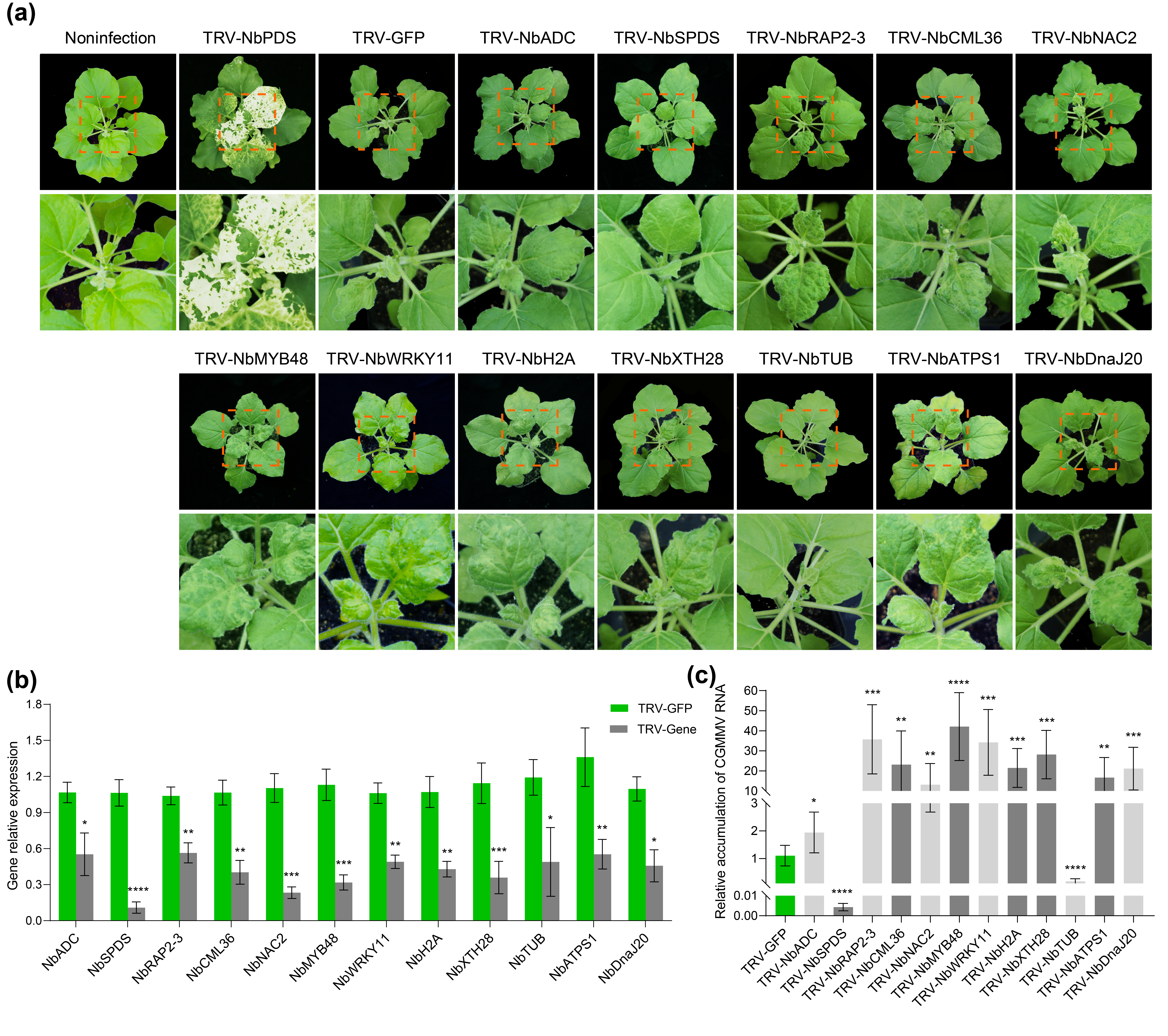

Supplement: Supplementary file 3 — FIGURE S3 The effects of individual silencing 12 homologues of watermelon genes on CGMMV infection by TRV‐based virus‐induced gene silencing (VIGS) systems in Nicotiana benthamiana. (a) Mosaic symptoms of various gene‐silenced leaves in N. benthamiana or controls at 9 days postinoculation (dpi) with CGMMV. (b) Gene silencing efficiency of TRV VIGS assays were determined through reverse transcription‐quantitative PCR (RT‐qPCR) (n = 9). (c) CGMMV accumulation from various gene‐silenced leaves in N. benthamiana or controls at 9 dpi with CGMMV were determined through RT‐qPCR (n = 9). The results were expressed as the mean ± SD, using a two‐tailed t test (*p < 0.05, **p < 0.01, ***p < 0.001, ****p < 0.0001) [file MPP-23-1361-s008.png]

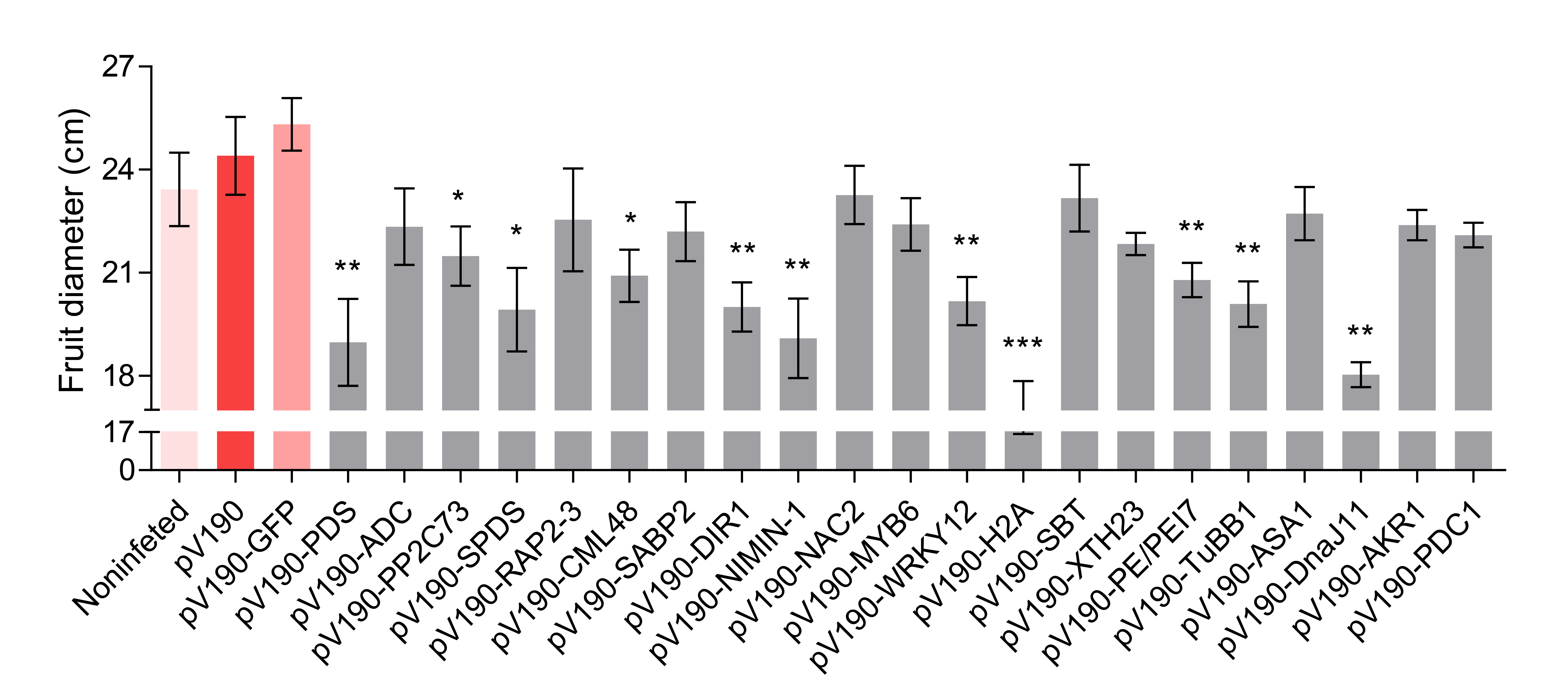

Supplement: Supplementary file 6 — FIGURE S6 Fruit diameter of gene‐silenced watermelons (n = 12). The results are expressed as the mean ± SD, using a two‐tailed t test (*p < 0.05, **p < 0.01, ***p < 0.001) [file MPP-23-1361-s009.png]
