# Supplementary material for: A novel strategy for improving watermelon resistance to cucumber green mottle mosaic virus by exogenous boron application
Source: Mol Plant Pathol. 2022 Jun 7;23(9):1361–80. doi: 10.1111/mpp.13234 (PMC9366068; doi:10.1111/mpp.13234)
Supplement: Supplementary file 16 — TABLE S10 Abbreviations of the genes and compounds in Figures 4 and 5 [file MPP-23-1361-s007.docx]

**Table S10 Abbreviations of the genes and compounds in Figure 4 and 5.**

| **Abbreviations of the genes and compounds in figure 4** | **Abbreviations of the genes and compounds in figure 5** |
| --- | --- |
| Orn, ornithine;  Put, putrescine;  Spd, spermidine;  Ac-Spd, acetylated spermidine;  Spm, spermine;  SAM, S-adenosylmethionine;  Dc-SAM, decarboxyl S-adenosylmethionine;  5’-MTA, 5’-methylthioadenosine;  Arg, arginine;  ADC, arginine decarboxylase;  SAMDC, S-adenosylmethionine decarboxylase;  SPDS, spermidine synthase;  SPMS, spermine synthase;  PAOX, polyamine oxidase;  Eth, ethylene;  SAM, S-adenosyl-L-methionine;  MET, L-Methionine;  ACC, 1-aminocyclopropane-1-carboxylic acid;  ACO, 1-aminocyclopropane-1-carboxylate oxidase;  ETR, ethylene receptor;  ERF, ethylene-responsive transcription factor;  ABA, abscisic acid;  ABA 8'-hydroxylase, abscisic acid 8'-hydroxylase;  PYR/PYL, ABA receptor PYR/PYL family;  PP2C, protein phosphatases type-2C;  SnRK2, sucrose nonfermenting 1-related protein kinases 2;  ABF, ABA-responsive element-binding factors;  IAA, indole-3-acetic acid;  Trp, tryptophan;  IPA, indole-3-pyruvate;  YUCCA, flavin monooxygenase;  LAX, auxin transporter;  TIR1, transport inhibitor response 1;  AUX/IAA, auxin-responsive protein;  ARF, auxin-responsive transcription factor;  CH3, Gretchen Hagen3;  SAUR, small auxin upregulated RNA;  5NG4, auxin-induced protein 5NG4. | CaM, calmodulin;  CML, calcium-binding protein;  SA, salicylic acid;  PO43, peroxidase 43;  RbohC, respiratory burst oxidase homolog protein C;  NIMIN, NIM1-INTERACTING;  NPR1, NON-EXPRESSOR OF PATHOGENESIS-RELATED GENES1;  TGA, transcription factor TGA;BON1, BONZAI1;  CBL, calcineurin B-like;  CIPK, CBL-interacting serine/threonine-protein kinase;  BRI1, BRASSINOSTEROID INSENSITIVE1;  NIK, NSP-interacting kinase;  BIK1, serine/threonine-protein kinase BIK1;  PBS1, serine/threonine-protein kinase PBS1-like;  RLCKVII, serine/threonine-protein kinase RLCKVII-like;  RBK1, receptor-like cytosolic serine/threonine-protein kinase RBK1;  OXI1, serine/threonine-protein kinase;  MAPK, mitogen-activated protein kinase;  DUSP, dual specificity protein phosphatase;  AP2/ERF, APETALA2/ethylene responsive factor;  bHLH, basic helix-loop-helix; MYB, (v-myb avian myeloblastosis viral oncogene homolog)-related transcription factor;  NAC, no apical meristem (NAM), Arabidopsis transcription activation factor (ATAF1/2), and cup-shaped cotyledon (CUC2);  WRKY, WRKY transcription factor;  bZIP, basic leucine zipper;  HD-Zip, homeodomain-leucine zipper;  TIFY, Jasmonate ZIM domain-containing protein,  TLP, thaumatin-like protein;  PR, pathogen-related;  CBSX5, CBS domain-containing protein CBSX5;  TRXh, thioredoxin H-type-like;  RGA2, disease resistance protein RGA2;  ABC transporter, ATP-binding cassette (ABC) transporters;  H2A, histone H2A;  ARP, apurinic endonuclease-redox protein;  UDG, uracil-DNA glycosylase;  DPL, deoxyribodipyrimidine photo-lyase;  RPA, replication protein A;  E3, E3 ubiquitin-protein ligase; defensin, defensin-like protein;  SBT, subtilisin-like protease;  NDR1, NON-RACE-SPECIFIC DISEASE RESISTANCE1;  HSPs, heat shock proteins;  GRAM, GRAM Domain–Containing Protein;  PLD p2, phospholipase D p2;  NB-LRR, nucleotide-binding domain and leucine-rich repeat;  Ser/Thr kinase, serine/threonine protein kinase;  SAR, systemic acquired resistance. |
